# Understanding the effect of educational attainment on the risk of retinal detachment: a Mendelian randomization mediation analysis

**DOI:** 10.1186/s40942-026-00869-4

**Published:** 2026-06-13

**Authors:** Andreas Katsimpris, Sebastian-Edgar Baumeister, Hansjörg Baurecht, Andrew Blaikie, Colin Goudie, Andrew J. Tatham, Michael Nolde

**Affiliations:** 1 Department of Ophthalmology, Princess Alexandra Eye Pavilion, University of Edinburgh, Edinburgh, EH3 9HA UK; 2 Institute of Health Services Research in Dentistry, University of Münster, Münster, Germany; 3 Department of Epidemiology and Preventive Medicine, University of Regensburg, Regensburg, Germany; 4 Global Health Team, School of Medicine, University of St Andrews, St Andrews, UK; 5 Centre for Clinical Brain Sciences, University of Edinburgh, Edinburgh, EH3 9HA UK

## Abstract

**Purpose:**

Higher educational attainment is generally associated with better health outcomes, but paradoxically, some evidence suggests an increased risk of rhegmatogenous retinal detachment (RD) among individuals with higher education, potentially due to myopia. This study aimed to investigate whether the association between education and RD is causal and to what extent it is mediated by refractive error and other modifiable lifestyle factors.

**Methods:**

We conducted univariable and multivariable Mendelian randomization (MR) analyses using publicly available genome-wide association study summary statistics from up to 3 million individuals of European ancestry. Genetically predicted educational attainment was examined for its effect on RD risk. MR mediation analysis was employed to estimate the contribution of refractive error, smoking, alcohol consumption, and body mass index (BMI) to the observed association.

**Results:**

Each one standard deviation increase in genetically predicted educational attainment was associated with a 1.50-fold higher risk of RD (95% confidence interval [CI]: 1.02–2.21; *p* = 0.04). MR mediation analysis suggested that the effect of education on RD was largely mediated by refractive error. Adjustments for smoking, alcohol use, and BMI showed limited impact on the association, reinforcing myopia as the principal mediator.

**Conclusions:**

Our findings provide evidence consistent with a possible causal effect between higher educational attainment and increased RD risk, driven predominantly by myopia. These results highlight the potential importance of refractive error as a pathway linking educational attainment and RD, which may be relevant for future prevention strategies.

**Supplementary Information:**

The online version contains supplementary material available at 10.1186/s40942-026-00869-4.

## Introduction

Lower education levels have been associated with a higher risk of several ocular diseases, like age-related macular degeneration and diabetic retinopathy [1]. This inverse relationship highlights the importance of socioeconomic factors and access to healthcare in the prevention and management of these conditions. Individuals with lower educational attainment often have limited access to information, preventive measures, and treatments that could mitigate the risk of such diseases [2].

However, rhegmatogenous retinal detachment (RD) may be an exception to this general trend. Contrary to many ocular diseases, a recent observational study has indicated that higher educational attainment is associated with an increased risk of RD [3]. The primary explanation for this paradoxical relationship is that higher education levels often correlate with higher risk and degree of myopia, a significant risk factor for RD [4]. Myopia is more prevalent among individuals who engage in extensive near-work activities such as reading and studying, which are common among those with higher education. Consequently, the increased prevalence of myopia in this population has been suggested to elevate their risk of RD.

While the associations between educational attainment and myopia, and between myopia and retinal detachment, are well documented, it remains unclear whether educational attainment has a causal effect on RD risk and to what extent this relationship is mediated through refractive error. Importantly, the extent to which RD risk could theoretically be reduced by intervening on downstream pathways such as myopia remains unknown. Addressing this question requires a causal inference framework that can disentangle direct and indirect effects, which cannot be achieved using conventional observational designs.

Thus, we employed Mendelian randomization (MR) to assess the association between educational attainment and RD. MR is an epidemiological method that employs genetic variants identified through genome-wide association studies (GWAS) as instrumental variables to assess the causal association between risk factors and diseases [5]. MR can also help to mitigate confounding factors and reverse causation, offering more reliable insights into the causal pathways linking educational attainment and RD. Additionally, we performed an MR mediation analysis to quantify the extent to which this effect is mediated through refractive error and other potential factors—including smoking, alcohol consumption, and body mass index (BMI) —in order to further understand how these factors interact. These variables were included to assess whether the association between educational attainment and RD could be explained by broader behavioral mechanisms, in contrast to a more specific biological pathway through refractive error.

## Materials and methods

### Study design

MR leverages genetic variants as instrumental variables to explore causal relationships between risk factors and diseases [5]. This approach reduces confounding and reverse causation since genetic variants are randomly assigned at conception, mimicking a randomized controlled trial [5]. In this study, we performed a two-sample MR using summary statistics of single-nucleotide polymorphisms (SNPs) from GWAS on educational attainment [6] and RD [7] to examine the effect of education on RD risk. We further aimed to estimate the proportion of education’s effect on RD mediated by refractive error, smoking, alcohol consumption, and BMI, using multivariable MR for mediation analysis [8]. These variables were included to represent potential alternative lifestyle-related pathways linking educational attainment to RD risk. Our research followed STROBE-MR guidelines [9] and “Guidelines for performing Mendelian randomization investigations” [10].

### Data sources

Genetic association estimates for educational attainment were sourced from a GWAS meta-analysis conducted by the Social Science Genetics Association Consortium, encompassing 3,037,499 individuals of European descent [11]. Educational attainment was quantified as the number of years of education, standardized across included studies using the International Standard Classification of Education. For the mediation MR analysis, we utilized summary statistics from GWAS of refractive error, smoking, alcohol consumption, and BMI. Genetic association estimates for refractive error were sourced from two GWAS analyses conducted using UK Biobank participants of European ancestry, aged 40 to 69 years [12], a population in which accommodative capacity is reduced, making noncycloplegic autorefraction a generally acceptable measure of refractive error. The first GWAS involved 95,619 participants who underwent noncycloplegic autorefraction to measure refractive error. The second GWAS included 287,448 participants who did not undergo autorefraction; instead, the refractive error was inferred from the age of onset of spectacle wear (AOSW), considering participants’ age, sex, and self-reported AOSW. These two GWAS datasets were combined using the METAL software [13], applying an inverse-variance weighted meta-analysis. This approach weights SNP-specific effect estimates by the inverse of their variance, thereby prioritizing more precise estimates. Although these phenotypes are defined differently, they have been shown to be strongly genetically correlated and are commonly combined in large-scale genetic studies to increase statistical power [12]. Genetic association estimates for smoking and alcohol consumption were sourced from the GWAS and Sequencing Consortium of Alcohol and Nicotine Use (GSCAN) [14]. The smoking GWAS involved 143,320 participants, whereas the alcohol consumption GWAS included 226,223 participants. In these datasets we excluded participants from UK Biobank and 23andMe to avoid sample overlap with our exposure GWAS. This study quantified smoking as the average number of cigarettes smoked per day by current or former smokers, while weekly alcohol drinks were calculated as the total number of alcoholic beverages consumed per week, considering all types of alcohol [14]. Genetic association estimates for BMI were obtained from the Genetic Investigation of Anthropometric Traits (GIANT) consortium’s GWAS meta-analysis, which included 806,834 individuals of European ancestry [15]. RD summary data were sourced from the FinnGen consortium’s R11 release, comprising 13,719 RD cases and 459,962 controls of European descent [7]. RD cases were identified based on ICD-9 or ICD-10 diagnostic criteria. Detailed information on population characteristics and specific trait definitions for these genetic association estimates, as well as, details regarding the genotyping, quality control, and imputation methods, can be found in the original publications [7, 11, 12, 14, 15].

### Selection of genetic variants as instrumental variables

We selected SNPs in the education GWAS that achieved genome-wide significance (P-value < 5*10^− 8^), following clumping for linkage disequilibrium (LD) at r^2^ < 0.001 over a 10 Mb window [10]. The MR-Steiger test was used to confirm the directionality of the causal relationship between education and RD [16]. SNPs more strongly correlated with the outcome than the exposure, as well as those showing influence in funnel and scatter plots, were excluded. Following this procedure, we selected 561 SNPs associated with educational attainment as instrumental variables. Finally, the proportion of variance in education explained by these SNPs was calculated through the summation of the coefficients of determination (R^2^).

### Statistical analysis

Following data harmonization based on HapMap3 [17], removal of strand-ambiguous variants, and alignment of association estimates, we calculated Wald ratios by dividing the logarithm of the odds ratio (Log OR) per allele for each SNP from the RD GWAS by its estimate from the educational attainment GWAS. The total effect of education on RD risk was evaluated using a multiplicative random effects inverse variance weighted (IVW) meta-analysis of the Wald ratios [18].

We tried to adhere to the three key MR assumptions: relevance, where genetic variants must be associated with the exposure; exchangeability, where instruments should not be related to confounders; and exclusion restriction, where instruments influence the outcome solely through the exposure [19]. The strength of our instrumental variables was calculated by assessing the F-statistic of the selected SNPs. We performed sensitivity analyses to identify any violations of our assumptions. This included testing for heterogeneity (Cochran’s Q and I_GX_^2^) to detect pleiotropy [20] and we applied several pleiotropy-robust MR methods, such as MR Egger regression, penalized weighted median, IVW radial regression, and MR-Pleiotropy Residual Sum and Outlier (MR-PRESSO) [21]. Additionally, a leave-one-out analysis determined if any single SNP influenced the IVW estimate.

**Fig. 1:**
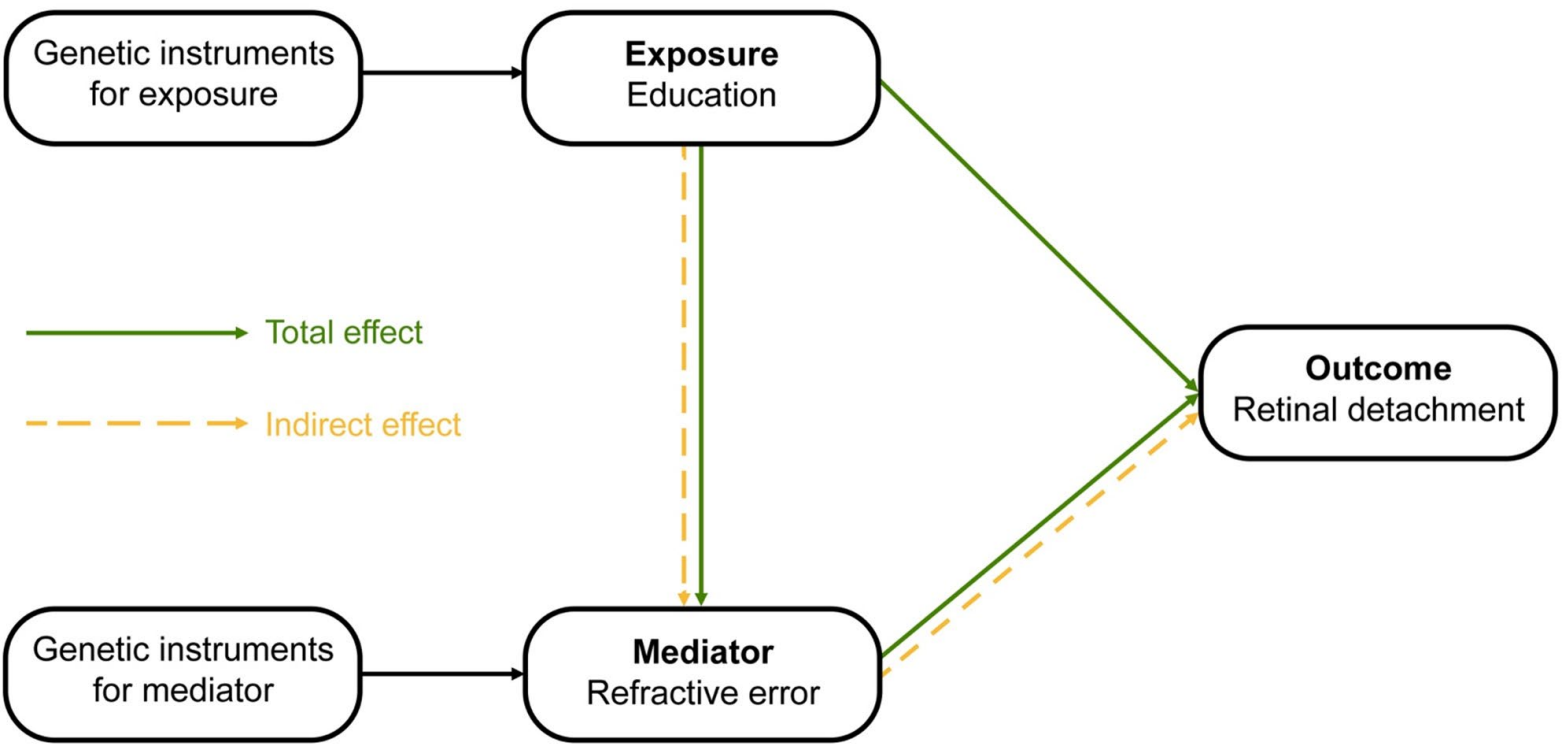
Directed acyclic graphs of the mediation analysis with Mendelian randomization *. * Univariable MR estimates the total effect of education on retinal detachment (RD), and multi- variable MR estimates the direct effect of education on RD conditional on the refractive error. The difference between these estimates gives the indirect effect of education on RD that is mediated via refractive error

To assess how education influences RD through refractive error, smoking, alcohol consumption, and BMI, we performed a multivariable MR mediation analysis as described in prior research [8]. This approach involves calculating two separate MR estimates: the total effect of education on RD using univariable MR, and the direct effect of education on RD, while adjusting for each mediator, using multivariable MR. The indirect effect of education on RD through each mediator was determined by calculating the difference between these estimates. Figure 1 presents a directed acyclic graph illustrating the mediating effect of refractive error on the relationship between education and RD. This model also applies to our other selected mediators: smoking, alcohol consumption, and BMI. We carried out a multivariable MR analysis to estimate the proportion of the total effect of education on RD that was mediated by each mediator individually and collectively. The mediated proportion was estimated on the log-odds scale as the indirect effect divided by the total effect. The indirect effect was calculated as the difference between the total effect from univariable MR and the direct effect from multivariable MR; thus, the mediated proportion was calculated as (log OR total effect − log OR direct effect) / log OR total effect. The proportions of mediation from individual multivariable MR analyses for each mediator may not sum up to 100%, given that the selected genetic instruments may be associated with a series of mediators along a causal pathway. To maintain the integrity of the MR mediation analysis, we ensured that SNPs chosen as instruments for education and mediators were non-overlapping across all GWAS datasets, preventing bias from SNP overlap [8].

All our association estimates are reported as OR per one standard deviation (SD) increase in educational attainment (corresponding to 3.4 years increase in years of education). We conducted all analyses with R version 4.2.1 [22] using the MVMR (0.3), TwoSampleMR (0.5.6), MendelianRandomization (0.5.1) and MRPRESSO (1.0) packages.

## Results

**Fig. 2:**
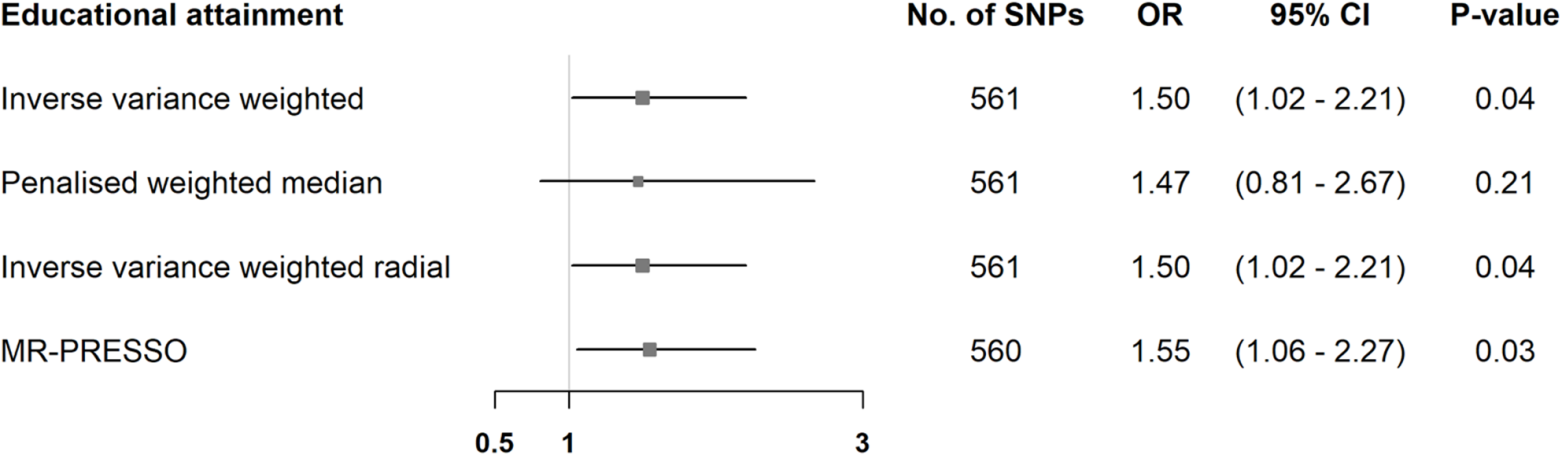
Mendelian randomization estimates for the effect of education on retinal detachment. Estimates are reported as changes in odds of retinal detachment per 1 standard deviation increase in educational attainment *. * SNP, single nucleotide polymorphism; CI, confidence interval; MR-PRESSO, Mendelian randomization pleiotropy residual sum and outlier

Supplementary Table 1 presents the phenotypic descriptive statistics for the studies included in the GWAS for exposure, mediator, and outcome variables. The average duration of education, weighted by sample size of the included studies, was 15.4 years with a SD of 3.4 years. The 561 SNPs selected from the educational attainment GWAS (Supplementary Fig. 1) accounted for 1.59% of the variability in educational attainment, with all SNPs displaying F-statistics of ≥ 29.5. Employing the IVW method, we found that higher genetically predicted educational attainment was associated with higher RD risk (OR = 1.50 per 1 SD increase in educational attainment; 95%CI = 1.02 to 2.21; P-value = 0.04) (Fig. 2). Results from pleiotropy-robust MR methods aligned with the IVW analysis estimate (Fig. 2).

The Wald ratios for the relationship between education and RD showed no heterogeneity, as indicated in Supplementary Table 2. The Cochran’s Q test for heterogeneity produced a value of 616.2 (p-value of 0.05). Furthermore, the MR-Egger analysis revealed no signs of directional pleiotropy, with intercepts not deviating from zero (Supplementary Table 3). The leave-one-SNP-out analysis also found that no single SNP significantly impacted the IVW estimate regarding the association between education and RD risk (Supplementary Table 4).

Figure 2 provides a visual representation of the MR mediation analysis concerning refractive error. Our results suggested that refractive error accounted for most of the observed effect (Table 1). Additionally, after adjusting for genetically predicted smoking, alcohol consumption, and BMI, the association between genetically predicted education and RD weakened (Table 1). Initially, the total effect on RD per 1 SD increase in educational attainment had an OR of 1.50 (95% CI: 1.02 to 2.21, p-value = 0.04). This effect decreased to 1.48 (95% CI: 1.02 to 2.14, p-value = 0.04) after adjusting for smoking, to 1.41 (95% CI: 0.99 to 2.03, p-value = 0.06) after adjusting for alcohol consumption, and to 1.38 (95% CI: 0.89 to 2.18, p-value = 0.16) after adjusting for BMI. When all mediators were included in the same model, the OR was attenuated to 0.73 (95% CI: 0.43 to 1.24, p-value = 0.25). The percentage reduction in the total effect due to adjustments was 100% for refractive error, 3.8% for smoking, 15.2% for alcohol consumption, and 20.5% for BMI. Combined, these mediators explained 100% of the total effect of genetically predicted educational attainment on RD risk.

**Table 1:** Direct effect of genetically predicted educational attainment on retinal detachment, after adjustment for mediators separately and together in the same multivariable MR model ^*^

	OR	(95% CI)	*P*-value	Proportion mediated
Total effect from univariable MR	1.50	(1.02 to 2.21)	0.04	-
Direct effect after adjustment for mediator				
Refractive error	0.74	(0.49 to 1.12)	0.16	100
Cigarettes per day	1.48	(1.02 to 2.14)	0.04	3.8
Drinks per week	1.41	(0.99 to 2.03)	0.06	15.2
Body mass index	1.38	(0.89 to 2.18)	0.16	20.5
All mediators	0.73	(0.43 to 1.24)	0.25	100

^*^ Odds ratio (OR) per 1 standard deviation increase in years of education. Proportion mediated (as a percentage) computed as indirect effect (logOR total effect – log OR direct effect)/total effect (log OR)

Abbreviation: CI, confidence interval

## Discussion

In this MR mediation analysis, we leveraged genetic data to assess the causal association between educational attainment and RD, while also examining the mediating role of refractive error, smoking, alcohol consumption, and BMI in this relationship. Our study extends existing knowledge by demonstrating, within a causal inference framework, that the relationship between educational attainment and RD is not merely associative but is largely explained through a specific mediating pathway involving refractive error. To our knowledge, this is the first study to quantify the potential causal effect of educational attainment on RD risk and to formally assess mediation through refractive error using MR methods. However, the estimated effect was modest in magnitude, with a relatively wide confidence interval and borderline statistical significance, and should therefore be interpreted with caution.

The association between educational attainment and myopia has been acknowledged for more than a century [23]. Recent research consistently identifies education as a significant risk factor for myopia development and progression [4, 24, 25]. In a case control study by Mountjoy et al., it was found that each additional year of education between ages 15 and 18 increases myopia risk by − 0.25 diopters annually, even after controlling for other factors, with a slower rate of progression after age 18 [4]. Additionally, the Global Myopia Prevalence and International Levels of Education study found a significant positive correlation between national educational performance, measured by PISA scores (Organisation for Economic Cooperation and Development Programme for International Student Assessment), and myopia prevalence in teenagers, with higher academic achievement linked to higher rates of myopia. Furthermore, there is a well-documented link of near-work activities and time spent outdoors with myopia development, although the exact mechanisms remain uncertain [26]. With the global emphasis on extended education, more time spent indoors, and near-work activities, an increase in retinal complications is also likely.

Despite the well-documented connection between education and myopia, there is limited evidence linking education to RD. Myopia is a major risk factor for developing RD [27] and since higher educational attainment is linked to a greater risk of myopia, it is plausible that education and RD are causally associated. One of the few studies to assess the association between education and RD is a case-control registry study involving 10,268 individuals from Sweden [3]. This recent case-control study supports this notion, revealing that individuals with higher educational levels have a significantly increased risk of RD indicating a potential link between prolonged education and higher RD risk. Specifically, the study found that individuals with postgraduate education had 77% higher odds of developing RD (OR = 1.77, 95% CI 1.19–2.64, *p* = 0.005) compared to those with only nine years of pre-upper secondary education. While this association aligns with our association estimates, it is important to note that the Swedish case-control study categorized education into seven levels, whereas our study measured education by the number of years completed.

Although higher education is associated with lower levels of smoking, alcohol consumption, and body mass index [28], our findings suggest that these variables do not substantially mediate the effect of education on RD. Instead, myopia emerges as the primary mediator in this association. Our results reinforce the notion that RD differs from other chronic ocular diseases such as age-related macular degeneration or diabetic retinopathy, where factors like smoking and body mass index are influential. Moreover, a crucial implication of our findings is the potential population-level impact of interventions targeting the education-myopia pathway. This distinction is important, as it suggests that the increased RD risk observed in more highly educated populations is not inherent to education itself, but is instead driven by a modifiable intermediate phenotype. Therefore, interventions aimed at preventing or slowing myopia progression may be relevant for reducing RD risk, although this cannot be directly inferred from the present analysis. As an MR study, our analysis provides insight into potential causal pathways but does not establish the effectiveness of specific interventions or quantify their impact on absolute risk. In addition, these findings refine the interpretation of risk by indicating that educational attainment itself is unlikely to be an independent causal factor for RD, but rather acts through a modifiable biological pathway. This has implications for risk communication and for prioritizing preventive strategies, suggesting that efforts should focus on early detection and control of myopia, particularly in populations exposed to prolonged educational intensity. This underscores the critical public health challenge of balancing high educational achievement with the risk of myopia and its complications, including RD, emphasizing the need for preventive strategies to mitigate these effects. The Global Myopia Prevalence and International Levels of Education study found a strong correlation between high educational outcomes and increased myopia prevalence [25]. However, it also identified that countries like Finland, United Kingdom and Australia, manage to achieve excellent educational results without a high prevalence of myopia. The educational policies of these countries could serve as exemplary models, as they consistently score high on PISA while maintaining relatively low myopia rates by having children spend less time on near work, more time outdoors, and starting formal education at a slightly later age.

This study’s primary strength is its innovative use of MR mediation analysis to assess the causal pathway linking education to RD. Another notable strength is the consistent association estimates derived from robust methods that account for pleiotropy, aligning closely with the IVW estimate and suggesting no model violations. However, there are several limitations that should be taken into account. Initially, our MR models operated under the assumption of a linear relationship between the identified risk factors and the observed outcomes, although the true association between education and RD may exhibit non-linear patterns. Secondly, weak instruments in two-sample MR can bias estimates towards observational results, especially with overlapping samples among the selected GWAS. In our univariable MR analysis of educational attainment and RD risk, no sample overlap was present. However, there was sample overlap in the datasets selected for multivariable MR. Since all the selected genetic instruments were strong (p-value < 5 × 10⁻⁸; F-statistic > 20), any potential bias is expected to be minimal. Thirdly, the analysis of education’s impact on RD did not incorporate time spent outdoors as a mediator due to sample overlap with the education GWAS. In addition, the interpretation of mediation results should be approached with caution, as MR-based mediation analyses rely on several assumptions, including the absence of horizontal pleiotropy and correct model specification, which may not be fully testable. Moreover, refractive error was assessed using a combination of noncycloplegic autorefraction and proxy measures based on AOSW, which may introduce measurement error, although these approaches are widely used and show strong genetic correlation with directly measured refractive error. In addition, because RD is a binary outcome, mediation proportions estimated on the OR scale may be affected by non-collapsibility, which can complicate interpretation. Therefore, the estimated mediated proportions should be interpreted cautiously and primarily as an indication of the degree of attenuation after adjustment for each mediator. Finally, caution is necessary when generalizing genetic associations from European populations to other ethnic groups, given potential variations specific to different populations.

In conclusion, our MR analyses suggest an elevated risk of RD associated with higher educational attainment. By utilizing MR mediation analysis, our findings additionally suggest that this effect appears to be largely mediated through myopia. This underscores the need to address myopia as a significant public health issue, particularly in highly educated populations. Preventive strategies, such as promoting outdoor activities and implementing interventions to control myopia progression, may play an important role in reducing the burden of RD, although further research is needed to establish their effectiveness.

## Supplementary Information

Below is the link to the electronic supplementary material.

Supplementary Material 1

## Data Availability

The summary statistics for the educational attainment GWAS are available at [https://thessgac.com] (access date: 2024/05/01). The retinal detachment summary statistics for the FinnGen GWAS are available at https://www.finngen.fi/en/access_results (access date: 2024/05/01). The refractive error summary statistics are available at https://www.ebi.ac.uk/gwas/publications/31670792 (access date: 2024/05/01). The smoking and alcohol consumption summary statistics are available at https://conservancy.umn.edu/handle/11299/201564 (access date: 2024/05/01). The body mass index summary statistics are available at https://zenodo.org/records/1251813 (access date: 2024/05/01).
