# Supplementary material for: Understanding the effect of educational attainment on the risk of retinal detachment: a Mendelian randomization mediation analysis
Source: Int J Retina Vitreous. 2026 Jun 13;12:85. doi: 10.1186/s40942-026-00869-4 (PMC13264816; doi:10.1186/s40942-026-00869-4)
Supplement: Supplementary file 1 — Supplementary Material 1 [file 40942_2026_869_MOESM1_ESM.docx]

**Educational attainment and the risk of retinal detachment: a Mendelian randomization mediation analysis**

­­­­­

**Authors:** Andreas Katsimpris, Sebastian-Edgar Baumeister, Hansjörg Baurecht, Andrew Blaikie , Colin Goudie, Andrew J Tatham, Michael Nolde

Supplementary Figures and Tables


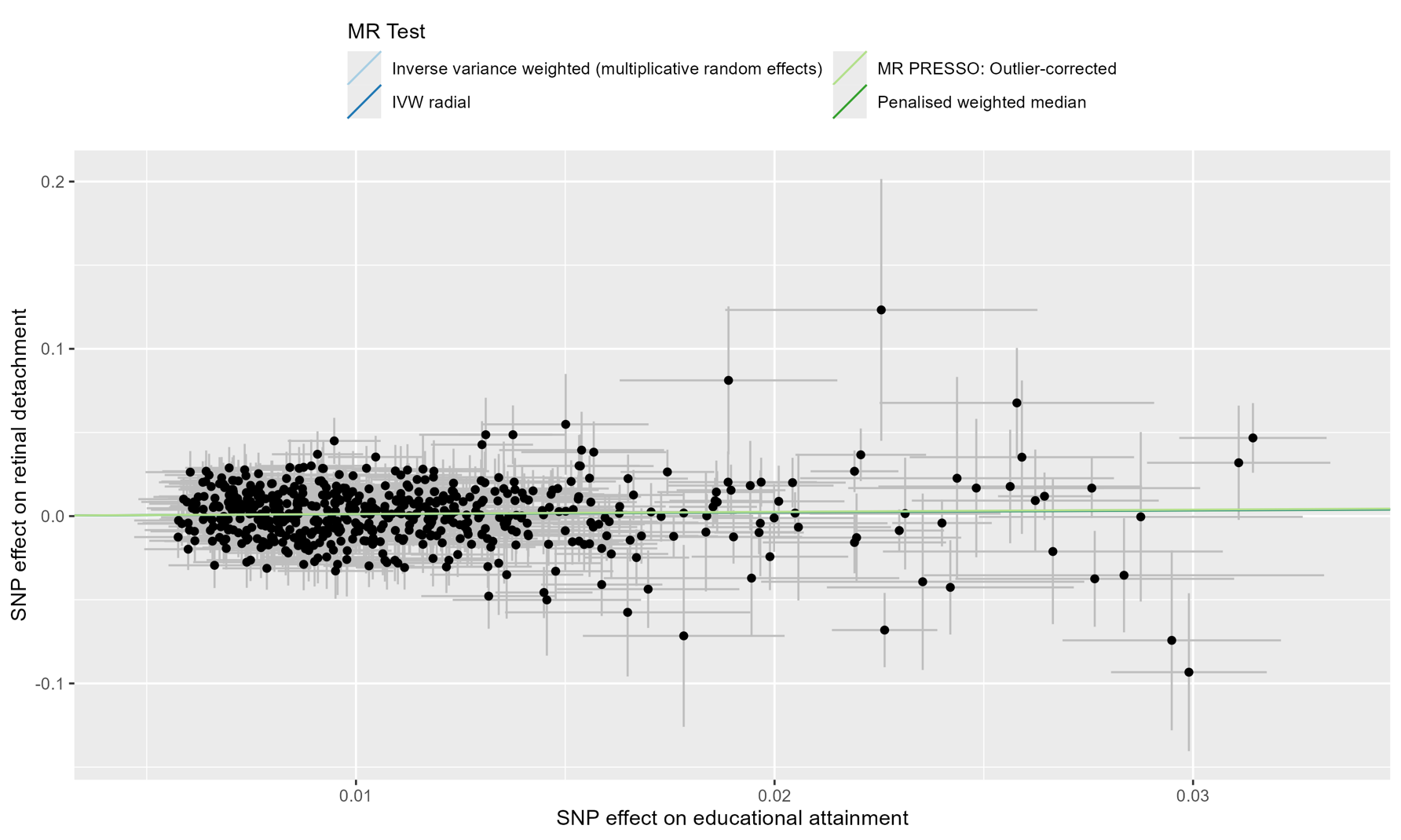
**Supplementary Figure 1** Scatter plot of SNP-educational attainment associations vs SNP- retinal detachment associations

SNP: single-nucleotide polymorphism. Lines for IVW radial, penalized weighted median and inverse variance weighted are not visible because they have the same slope with MR PRESSO line.

**Supplementary Table 1** Phenotypic descriptive statistics of studies included in the exposure, mediator, and outcome genome-wide association studies ^a^

| GWAS / Study | Phenotype | | | | | | | | |
| --- | --- | --- | --- | --- | --- | --- | --- | --- | --- |
| (Okbay et al., 2022) | Educational attainment | | | | | | | | |
|  | N | | Birth year (mean) | | Female (%) | | | Education years (Mean, SD) | |
| 23andMe | 2,272,216 | | 1969 | | 56 | | | 15.71 (2.65) | |
| All Health | 5,690 | | 1979 | | 53 | | | 15.6 (2.8) | |
| EGCUT | 8,972 | | 1956 | | 68 | | | 12.7 (4.3) | |
| ELSA | 6,065 | | 1940 | | 53 | | | 9.5 (7.0) | |
| FENLAND | 8,535 | | 1961 | | 53 | | | 15.3 (3.6) | |
| Geisinger | 14,562 | | 1960 | | 71 | | | 14.8 (3.0) | |
| GSII | 15,941 | | 1957 | | 60 | | | 13.3 (3.2) | |
| NORFOLK | 19,193 | | 1936 | | 53 | | | 13.7 (2.9) | |
| UKB | 441,121 | | 1951 | | 54 | | | 15.23 (5.55) | |
| UKHLS | 8,094 | | 1954 | | 56 | | | 12.9 (3.9) | |
| VIKING | 1,841 | | 1961 | | 59 | | | 13.6 (3.5) | |
| WLS | 7,789 | | 1938 | | 52 | | | 15.8 (3.1) | |
| ACPRC | 1,713 | | 1923 | | 71 | | | 14.8 (4.0) | |
| AGES | 3,212 | | 1927 | | 58 | | | 13.6 (4.9) | |
| ALSPAC | 2,877 | | 1959 | | 100 | | | 14.3 (4.0) | |
| ASPS | 777 | | 1932 | | 57 | | | 11.6 (2.9) | |
| BASE-II | 1,619 | | 1948 | | 52 | | | 16.8 (3.1) | |
| CoLaus | 3,269 | | 1950 | | 53 | | | 15.0 (3.5) | |
| COPSAC2000 | 318 | | 1966 | | 47 | | | 17.3 (3.6) | |
| CROATIA-Korčula | 842 | | 1950 | | 64 | | | 12.5 (3.3) | |
| deCODE | 46,758 | | 1945 | | 57 | | | 13.4 (3.3) | |
| DHS | 953 | | 1949 | | 53 | | | 13.8 (3.3) | |
| DIL | 2,578 | | 1958 | | 52 | | | 14.0 (4.5) | |
| ERF | 2,433 | | 1952 | | 55 | | | 10.2 (3.4) | |
| FamHS | 3,483 | | 1941 | | 53 | | | 15.6 (2.8) | |
| FINRISK | 1,685 | | 1946 | | 46 | | | 13.8 (5.1) | |
| FTC | 2,418 | | 1945 | | 56 | | | 11.9 (3.4) | |
| GOYA | 1,459 | | 1947 | | 0 | | | 14.4 (4.6) | |
| GRAPHIC | 727 | | 1951 | | 53 | | | 14.9 (3.1) | |
| GS | 8,776 | | 1955 | | 59 | | | 14.0 (4.1) | |
| H2000 Cases | 797 | | 1949 | | 50 | | | 12.2 (4.1) | |
| H2000 Controls | 819 | | 1949 | | 52 | | | 12.7 (4.2) | |
| HBCS | 1,617 | | 1941 | | 57 | | | 11.7 (4.5) | |
| HCS | 1,946 | | 1940 | | 49 | | | 14.2 (3.3) | |
| HNRS (CorexB) | 1,401 | | 1942 | | 50 | | | 14.4 (3.5) | |
| HNRS (Oexpr) | 1,347 | | 1942 | | 50 | | | 15.2 (3.6) | |
| HNRS (Omni1) | 778 | | 1942 | | 52 | | | 14.9 (3.6) | |
| HRS | 9,963 | | 1940 | | 42 | | | 14.3 (3.2) | |
| Hypergenes | 815 | | 1945 | | 46 | | | 12.3 (3.7) | |
| INGI-CARL | 947 | | 1946 | | 58 | | | 9.7 (3.4) | |
| INGI-FVG | 943 | | 1951 | | 60 | | | 9.5 (2.6) | |
| KORA S3 | 2,655 | | 1945 | | 51 | | | 13.7 (2.6) | |
| KORA S4 | 2,721 | | 1949 | | 51 | | | 14.1 (2.8) | |
| LBC1921 | 515 | | 1921 | | 58 | | | 11.3 (1.8) | |
| LBC1936 | 1,003 | | 1936 | | 49 | | | 13.2 (3.7) | |
| LifeLines | 12,539 | | 1960 | | 58 | | | 13.5 (4.2) | |
| MCTFR | 3,819 | | 1953 | | 54 | | | 15.9 (3.3) | |
| MGS | 2,313 | | 1951 | | 50 | | | 15.6 (3.2) | |
| MoBa | 622 | | 1971 | | 100 | | | 17.6 (4.0) | |
| NBS | 1,808 | | 1941 | | 50 | | | 12.9 (4.3) | |
| NESDA | 1,820 | | 1958 | | 64 | | | 14.2 (4.9) | |
| NFBC66 | 5,297 | | 1966 | | 52 | | | 13.9 (2.4) | |
| NTR | 5,246 | | 1958 | | 64 | | | 14.5 (4.9) | |
| OGP | 370 | | 1950 | | 0 | | | 10.0 (3.5) | |
| OGP-Talana | 544 | | 1949 | | 59 | | | 9.1 (3.1) | |
| ORCADES | 1,828 | | 1952 | | 60 | | | 13.0 (3.3) | |
| PREVEND | 3,578 | | 1948 | | 48 | | | 12.0 (4.7) | |
| QIMR | 8,006 | | 1956 | | 59 | | | 17.3 (3.2) | |
| RS-I | 6,108 | | 1922 | | 60 | | | 10.4 (3.5) | |
| RS-II | 1,667 | | 1935 | | 52 | | | 12.5 (3.7) | |
| RS-III | 3,040 | | 1950 | | 56 | | | 12.9 (4.2) | |
| Rush-MAP | 887 | | 1921 | | 72 | | | 13.3 (2.9) | |
| Rush-ROS | 808 | | 1921 | | 66 | | | 16.6 (3.3) | |
| SardiNIA | 5,616 | | 1955 | | 58 | | | 10.4 (4.2) | |
| SHIP | 3,556 | | 1945 | | 50 | | | 12.8 (3.7) | |
| SHIP-TREND | 901 | | 1956 | | 57 | | | 14.5 (2.7) | |
| STR – Salty | 4,832 | | 1951 | | 52 | | | 14.3 (3.7) | |
| STR – Twingene | 9,553 | | 1941 | | 53 | | | 12.9 (4.2) | |
| THISEAS | 829 | | 1950 | | 33 | | | 13.2 (3.1) | |
| TwinsUK | 4,012 | | 1949 | | 100 | | | 12.9 (4.8) | |
| WTCCC58C | 2,804 | | 1958 | | 48 | | | 14.1 (4.5) | |
| YFS | 2,029 | | 1969 | | 55 | | | 15.8 (3.0) | |
| ACPRC | 1,713 | | 1961 | | 71 | | | 14.8 (4.0) | |
| (Ghorbani Mojarrad et al., 2020) | | Refractive error | | | | | | | |
|  | | N | |  |  | | |  | |
| Autorefraction-measured MSE | | 95,619 | |  |  | | |  | |
| AOSW-inferred MSE | | 287,448 | |  |  | | |  | |
| (Liu et al., 2019) | Smoking and alcohol consumption | | | | | | | | |
|  | N | | Cigarettes per day (Mean, SD) | | N | | Drinks per week (Mean, SD) |  | |
| ALSPAC | 4,314 | | 3.4 (1.6) | | 8,913 | | 5.5 (2.7) |  | |
| ARIC | 5,689 | | 3.0 (1.1) | | 6,250 | | 6.0 (3.0) |  | |
| BLS | 546 | | 0.7 (0.8) | | - | | - |  | |
| CAAD | 523 | | 2.4 (1.2) | | 916 | | 8.9 (3.0) |  | |
| COGEND | 1,940 | | 2.6 (1.4) | | 1,924 | | 19.3 (5.0) |  | |
| COPDGene | 6,613 | | 3.4 (0.9) | | - | | - |  | |
| deCODE | 44,505 | | 0.9 (0.8) | | 36,818 | | 6.7 (2.5) |  | |
| EGCUT | 5633 | | 2.2 (0.9) | | 10,667 | | 6 (4.4) |  | |
| FHS | 3,065 | | 2.7 (1.1) | | 5,105 | | 7.8 (3.1) |  | |
| FinnTwin | 498 | | 2.2 (0.8) | | 969 | | 9.9 (3.0) |  | |
| GERA | - | | - | | 47,967 | | 7.3 (2.6) |  | |
| GFG | 835 | | 2.2 (0.9) | | 1,365 | | 4.7 (2.2) |  | |
| Harvard | 5,271 | | 2.8 (1.2) | | 9,296 | | 7.0 (3.6) |  | |
| HRS | 5,306 | | 2.9 (1.3) | | 5,635 | | 5.5 (2.9) |  | |
| HUNT | 33,705 | | 2.1 (0.7) | | 47,325 | | 2.9 (2.8) |  | |
| MCTFR | 2,535 | | 2.6 (1.5) | | 3,899 | | 4.5 (2.1) |  | |
| MESA | 1,150 | | 2.8 (1.2) | | 1,632 | | 4 (2.5) |  | |
| METSIM | 1,507 | | 2.3 (0.8) | | 7,403 | | 10.5 (3.3) |  | |
| NESCOG | 210 | | 2.1 (0.9) | | 429 | | 9.5 (3.9) |  | |
| NAG-FIN | 1,716 | | 2.8 (0.9) | | 1,697 | | 15.6 (5.1) |  | |
| NTR | 2,725 | | 2.4 (0.9) | | 5,084 | | 9.5 (3.4) |  | |
| OZALC | 4,409 | | 3.7 (1.1) | | 9,947 | | 11.6 (4.0) |  | |
| SardiNIA | 2,105 | | 2.5 (1.1) | | 2,570 | | 9.5 (3.1) |  | |
| WHI | 8,410 | | 3.6 (1.3) | | 4,696 | | 4.3 (3.0) |  | |
| (Pulit et al., 2018) | Body mass index | | | | | | | | |
|  | N | | Mean, SD | | Female (%) | |  |  |  |
| UKB + GIANT | 806,834 | | 27.4 (4.8) | | 54 | |  |  |  |
| (Kurki et al., 2023) | Retinal detachment | | | | | | | | |
|  | N | | Female % | |  | | Median age at first event | Retinal detahcment % | |
| FINNGEN | 453,733 | | 56.1 | |  |  | 63.1 | 3.0 | |

^a^ Self-reported household income was collected using a 5 point scale corresponding to the total household income before tax, 1 being less than £18,000, 2 being £18,000 - £29,999, 3 being £30,000 - £51,999, 4 being £52,000 – £100,000, and 5 being greater than £100,000.

GWAS, genome-wide association study. 23andMe, 23andMe Inc.

ACPRC, Manchester Studies of Cognitive Ageing. AGES. Age, Gene/ Environment Susceptibility–Reykjavik Study. Add Health, National Longitudinal Study of Adolescent to Adult Health. ADVANCE-CAD, Atherosclerotic Disease, VAscular FuNction, & GenetiC Epidemiology Study. ALSPAC, Avon Longitudinal Study of Parents and Children. ARIC, Atherosclerosis Risk in Communities. BLTS, Brisbane Longitudinal Twin Study. ASPS, Austrian Stroke Prevention Study. BASE-II, Berlin Aging Study II. B1958C, 1958 British Birth Cohort. BHS, Busselton Health Study. CADD, Center on Antisocial Drug Dependence. CLHNS, Cebu Longitudinal Health and Nutrition Survey (Offspring). COGEND, Collaborative Genetic Study of Nicotine Dependence. COPDGene, Genetics of Chronic Obstructive Pulmonary Disease. CoLaus, Cohorte Lausannoise. COPSAC2000, Copenhagen Studies on Asthma in Childhood 2000. COROGENE, Genetic Predisposition of Coronary Heart Disease in Patients Verified with Coronary Angiogram. CROATIA-Korčula, Croatia Korčula. 2D 2007, FIN-D2D study 2007 cohort. deCODE, deCODE Genetics. DIAGEN, The DIAbetes GENetics Study. DHS, Dortmund Health Study. DIL, Wellcome Trust Diabetes and Inflammation Laboratory. DESIR, Data from an Epidemiological Study on the Insulin Resistance syndrome. DILGOM, Dietary, life style, and genetic determinants of obesity and metabolic syndrome. DPS, Diabetes Prevention Study. EGCUT, Estonian Genome Center. ELSA, English Longitudinal Study of Ageing. ERF, Erasmus Rucphen Family Study. ELY, MRC Ely Study. EMIL, Echinococcus Multilocularis and Internal Diseases in Leutkirch (EMIL) study SWABIA. EPIC, European Prospective Investigation into Cancer. FBPP, Family Blood Pressure Program: GenNet and HyperGEN studies. FamHS, Family Heart Study. FENLAND, The Fenland Study. FINRISK, The National FINRISK Study. FTC, Finnish Twin Cohort. FHS, Framingham Heart Study. Geisinger, Geisinger Health System.GERA, Genetic Epidemiology Research in Adult Health and Aging. GFG, Genes for Good. Harvard, Nurses' Health Study, Nurses’ Health Study II, and Health Professionals' Follow-up Study. HFPS, Health Professionals Follow-up Study. GSII. Generation Scotland: Scottish Family Health Study. GOYA, Genetics of Overweight Young Adults. GOOD, . Gothenburg Osteoporosis and Obesity Determinants Study. GXE, Kingston Gene-by-environment. GRAPHIC, Genetic Regulation of Arterial Pressure in Humans. GS, Generation Scotland. H2000, Health 2000. HBCS, Helsinki Birth Cohort Study. HCS, Hunter Community Study. Health ABC, Health, Aging, and Body Composition Study. HERITAGE, Health, Risk Factors, Training and Genetics (HERITAGE) Family Study. HNRS, Heinz Nixdorf Recall Study. HRS, Health and Retirement Study. HUNT, Nord-Trøndelag Health Study. Hypergenes, Hypergenes. InCHIANTI, Invecchiare in Chianti. INGI-CARL, Italian Network of Genetic Isolates – Carlantino. INGI-FVG, Italian Network of Genetic Isolates - Friuli Venezia Giulia. KORA, Kooperative Gesundheitsforschung in der Region Augsburg. LBC, Lothian Birth Cohort. LifeLines, The LifeLines Cohort Study. LLS, Leiden Longevity Study. LOLIPOP, London Life Sciences Prospective Population Study. LURIC, Ludwigshafen Risk and Cardiovascular Health Study. MCTFR, Minnesota Center for Twin and Family Research.. METSIM, Metabolic Syndrome in Men. MGS, Molecular Genetics of Schizophrenia. MoBa, Mother and Child Cohort of NIPH. METSIM, Metabolic Syndrome In Men. MORGAM, MOnica Risk, Genetics, Archiving and Monograph. NBS, Nijmegen Biomedical Study. NESDA, Netherlands Study of Depression and Anxiety. NFBC66, Northern Finland Birth Cohort 1966. NSHD, MRC National Survey of Health & Development. NESCOG, Netherlands Study on Cognition, Environment and Genes. NAG-FIN, Nicotine Addiction Genetics-Finland. NORFOLK, EPIC-Norfolk Prospective Population Study..NTR, Netherlands Twin Register. OGP, Ogliastra Genetic Park. OGP-Talana, Ogliastra Genetic Park-Talana. ORCADES, Orkney Complex Disease Study. OZALC, Australian Twin-Family Studies on Nicotine and Alcohol Genetics. PIVUS, Prospective Investigation of the Vasculature in Uppsala Seniors. PREVEND, Prevention of Renal and Vascular End-stage Disease. QIMR, Queensland Institute of Medical Research. PROCARDIS, Precocious Coronary Artery Disease. QFS, Quebec Family Study. PROMIS, Pakistan Risk of Myocardial Infarction Study. RISC, Relationship between Insulin Sensitivity and Cardiovascular disease Study. RS, Rotterdam Study. Rush-MAP, Rush University Medical Center - Memory and Aging Project. Rush-ROS, Rush University Medical Center - Religious Orders Study. SardiNIA, SardiNIA project. SHIP, Study of Health in Pomerania. SHIP-Trend, Study of Health in Pomerania Trend. SCARFSHEEP, Stockholm Coronary Artery Risk Factors/Stockholm Heart Epidemiology Programme. SORBS, Self-contained population from Eastern Germany, European Descent. SPT, Spanish Town; subset of International Collaborative Study of Hypertension in Blacks (ICSHIB). STR, Swedish Twin Registry. TANDEM, Seychelles Family Study (TANDEM). . THISEAS, The Hellenic Study of Interactions between SNPs & Eating in Atherosclerosis Susceptibility. TRAILS, Tracking Adolescents' Individual Lives Survey. TROSMSO, Tromsø study. TwinsUK, St Thomas’ UK Adult Twin Registry. UKB, UK Biobank. UKHLS, The UK Household Longitudinal Study. ULSAM, Uppsala Longitudinal Study of Adult Men. WHI, Women’s Health Initiative.. WTCCC, Wellcome Trust Case Control Consortium. VIKING, Viking Health Study – Shetland. WLS, Wisconsin Longitudinal Study. WTCCC58C, 1958 British Birth Cohort. YFS, The Cardiovascular Risk in Young Finns Study.

**Supplementary Table 2** Associations of single nucleotide polymorphisms for educational attainment

|  |  |  |  | Estimates for educational attainment | | | | Estimates for retinal detachment | | |
| --- | --- | --- | --- | --- | --- | --- | --- | --- | --- | --- |
| SNP | EA | OA | EAF | BETA | SE | P | F | BETA | SE | P |
| rs10014934 | T | C | 0.611 | 0.009 | 0.001 | 3.1e-17 | 71.5 | -0.016 | 0.013 | 0.206 |
| rs10016110 | A | G | 0.370 | 0.009 | 0.001 | 1.4e-18 | 76.9 | 0.013 | 0.013 | 0.302 |
| rs10063055 | T | C | 0.230 | -0.012 | 0.001 | 3.3e-24 | 103.1 | 0.013 | 0.015 | 0.355 |
| rs10167909 | T | C | 0.197 | 0.009 | 0.001 | 2.0e-12 | 49.9 | -0.009 | 0.016 | 0.546 |
| rs10180485 | T | G | 0.883 | 0.012 | 0.002 | 1.3e-13 | 55.1 | 0.002 | 0.019 | 0.903 |
| rs10180845 | T | C | 0.656 | -0.010 | 0.001 | 1.0e-18 | 77.6 | 0.007 | 0.013 | 0.607 |
| rs10186870 | T | G | 0.606 | -0.006 | 0.001 | 1.9e-08 | 31.7 | 0.008 | 0.013 | 0.516 |
| rs10189857 | A | G | 0.563 | 0.014 | 0.001 | 7.7e-39 | 169.0 | 0.020 | 0.012 | 0.101 |
| rs10193498 | A | T | 0.731 | 0.011 | 0.001 | 7.9e-21 | 88.2 | -0.008 | 0.015 | 0.566 |
| rs10202640 | A | G | 0.196 | -0.010 | 0.001 | 8.2e-15 | 60.5 | 0.006 | 0.017 | 0.711 |
| rs10240905 | T | C | 0.374 | -0.011 | 0.001 | 3.0e-23 | 98.0 | -0.012 | 0.014 | 0.363 |
| rs10256396 | A | C | 0.355 | 0.007 | 0.001 | 3.3e-09 | 34.7 | 0.018 | 0.013 | 0.168 |
| rs10283803 | T | C | 0.531 | -0.006 | 0.001 | 3.9e-08 | 30.0 | 0.013 | 0.012 | 0.315 |
| rs10401329 | A | G | 0.137 | 0.011 | 0.002 | 1.2e-11 | 46.2 | -0.009 | 0.018 | 0.634 |
| rs1043595 | A | G | 0.261 | 0.017 | 0.001 | 2.4e-45 | 198.6 | 0.013 | 0.015 | 0.399 |
| rs10481106 | A | G | 0.156 | -0.012 | 0.001 | 8.9e-18 | 73.8 | -0.003 | 0.016 | 0.860 |
| rs10496632 | C | G | 0.288 | 0.011 | 0.001 | 8.3e-22 | 92.2 | -0.014 | 0.013 | 0.311 |
| rs10507085 | T | C | 0.068 | -0.012 | 0.002 | 9.1e-10 | 37.5 | -0.010 | 0.020 | 0.616 |
| rs1056010 | T | C | 0.587 | -0.010 | 0.001 | 1.2e-20 | 87.0 | -0.004 | 0.013 | 0.755 |
| rs1058790 | A | G | 0.806 | -0.014 | 0.001 | 1.4e-24 | 105.4 | 0.000 | 0.019 | 0.994 |
| rs10732635 | T | G | 0.551 | 0.007 | 0.001 | 3.8e-11 | 44.1 | 0.013 | 0.013 | 0.323 |
| rs10734924 | T | G | 0.257 | -0.012 | 0.001 | 4.8e-23 | 97.7 | -0.009 | 0.015 | 0.568 |
| rs10739569 | C | G | 0.696 | -0.007 | 0.001 | 2.5e-10 | 40.2 | 0.002 | 0.014 | 0.912 |
| rs10740572 | A | T | 0.778 | -0.008 | 0.001 | 1.9e-11 | 44.9 | -0.015 | 0.014 | 0.305 |
| rs10745307 | A | G | 0.565 | -0.011 | 0.001 | 2.8e-24 | 103.7 | 0.026 | 0.013 | 0.037 |
| rs10748559 | T | C | 0.624 | -0.007 | 0.001 | 1.6e-09 | 36.2 | 0.002 | 0.013 | 0.864 |
| rs10752262 | T | C | 0.409 | 0.009 | 0.001 | 2.1e-18 | 77.3 | -0.010 | 0.013 | 0.421 |
| rs10763979 | A | G | 0.615 | 0.009 | 0.001 | 1.5e-17 | 72.6 | 0.029 | 0.013 | 0.025 |
| rs10765789 | A | G | 0.369 | 0.011 | 0.001 | 1.3e-23 | 99.6 | -0.001 | 0.014 | 0.938 |
| rs10772644 | C | G | 0.887 | 0.014 | 0.002 | 1.1e-16 | 69.1 | 0.014 | 0.017 | 0.409 |
| rs10815961 | T | C | 0.491 | 0.006 | 0.001 | 1.3e-09 | 36.6 | -0.002 | 0.012 | 0.868 |
| rs10819974 | T | C | 0.223 | -0.008 | 0.001 | 4.0e-11 | 43.9 | -0.004 | 0.014 | 0.774 |
| rs10835389 | T | C | 0.642 | -0.010 | 0.001 | 4.0e-18 | 74.7 | -0.007 | 0.013 | 0.574 |
| rs10853981 | A | G | 0.325 | -0.009 | 0.001 | 6.6e-15 | 61.1 | -0.011 | 0.014 | 0.423 |
| rs10865834 | A | G | 0.254 | -0.010 | 0.001 | 3.6e-18 | 75.2 | -0.004 | 0.015 | 0.807 |
| rs10867548 | A | G | 0.861 | 0.009 | 0.001 | 4.3e-10 | 39.0 | -0.024 | 0.019 | 0.201 |
| rs10874759 | A | G | 0.653 | 0.013 | 0.001 | 1.1e-31 | 137.3 | 0.006 | 0.013 | 0.627 |
| rs10887465 | A | C | 0.275 | 0.013 | 0.001 | 4.9e-27 | 116.9 | -0.017 | 0.013 | 0.205 |
| rs10887592 | A | G | 0.256 | 0.007 | 0.001 | 3.3e-08 | 30.5 | -0.009 | 0.013 | 0.526 |
| rs10895719 | T | C | 0.778 | -0.011 | 0.001 | 6.6e-17 | 69.6 | -0.004 | 0.014 | 0.767 |
| rs10929474 | A | T | 0.225 | 0.011 | 0.001 | 5.1e-19 | 80.0 | 0.001 | 0.015 | 0.921 |
| rs11019128 | T | C | 0.359 | -0.009 | 0.001 | 6.3e-16 | 65.0 | 0.013 | 0.013 | 0.318 |
| rs11028323 | A | C | 0.515 | -0.010 | 0.001 | 6.2e-21 | 87.8 | -0.013 | 0.012 | 0.291 |
| rs11081529 | T | C | 0.725 | 0.012 | 0.001 | 7.1e-26 | 110.2 | 0.001 | 0.013 | 0.931 |
| rs11138947 | T | C | 0.712 | 0.010 | 0.001 | 2.2e-16 | 67.0 | -0.009 | 0.014 | 0.506 |
| rs11157931 | A | C | 0.397 | -0.012 | 0.001 | 6.0e-27 | 115.9 | 0.011 | 0.013 | 0.402 |
| rs11158200 | A | G | 0.540 | 0.009 | 0.001 | 2.5e-16 | 67.2 | 0.005 | 0.012 | 0.716 |
| rs11159067 | A | G | 0.658 | 0.009 | 0.001 | 1.8e-17 | 72.9 | 0.045 | 0.014 | 0.001 |
| rs11172371 | T | C | 0.314 | -0.011 | 0.001 | 1.2e-21 | 91.3 | -0.018 | 0.013 | 0.170 |
| rs11181124 | T | C | 0.797 | -0.008 | 0.001 | 1.1e-10 | 41.5 | 0.016 | 0.015 | 0.289 |
| rs111821073 | T | C | 0.149 | 0.015 | 0.001 | 4.7e-26 | 111.8 | 0.030 | 0.018 | 0.102 |
| rs11191193 | A | G | 0.673 | 0.016 | 0.001 | 1.4e-49 | 219.6 | 0.006 | 0.013 | 0.658 |
| rs11196397 | A | C | 0.262 | 0.009 | 0.001 | 2.2e-14 | 58.8 | 0.006 | 0.014 | 0.662 |
| rs11218422 | T | C | 0.566 | 0.009 | 0.001 | 1.1e-16 | 68.8 | -0.009 | 0.012 | 0.488 |
| rs112255786 | C | G | 0.930 | -0.017 | 0.002 | 6.5e-15 | 60.7 | 0.044 | 0.023 | 0.060 |
| rs11245450 | A | G | 0.420 | 0.007 | 0.001 | 1.1e-10 | 41.9 | 0.017 | 0.012 | 0.172 |
| rs1125854 | T | C | 0.361 | -0.008 | 0.001 | 9.0e-13 | 51.2 | -0.001 | 0.013 | 0.922 |
| rs11264531 | T | C | 0.363 | 0.007 | 0.001 | 3.0e-10 | 39.8 | 0.018 | 0.013 | 0.172 |
| rs112780312 | A | G | 0.284 | -0.010 | 0.001 | 1.1e-16 | 68.5 | -0.025 | 0.013 | 0.066 |
| rs1128687 | T | C | 0.592 | -0.008 | 0.001 | 1.8e-14 | 58.8 | 0.003 | 0.013 | 0.799 |
| rs1128956 | T | G | 0.819 | -0.010 | 0.001 | 1.5e-13 | 54.3 | 0.015 | 0.019 | 0.443 |
| rs113389222 | T | C | 0.114 | -0.012 | 0.002 | 2.7e-13 | 53.5 | 0.008 | 0.018 | 0.672 |
| rs114440021 | T | C | 0.111 | 0.012 | 0.002 | 7.0e-14 | 55.8 | 0.020 | 0.021 | 0.338 |
| rs114468556 | A | T | 0.037 | 0.029 | 0.003 | 1.2e-29 | 127.7 | -0.074 | 0.054 | 0.167 |
| rs115003386 | C | G | 0.051 | 0.013 | 0.002 | 3.8e-08 | 30.2 | -0.028 | 0.031 | 0.364 |
| rs115124024 | A | C | 0.953 | 0.018 | 0.002 | 1.5e-13 | 54.7 | -0.072 | 0.054 | 0.188 |
| rs1156541 | T | C | 0.763 | 0.007 | 0.001 | 1.5e-08 | 31.8 | 0.021 | 0.014 | 0.123 |
| rs11571404 | T | C | 0.176 | 0.009 | 0.001 | 3.3e-11 | 43.9 | -0.029 | 0.016 | 0.063 |
| rs116068530 | A | G | 0.020 | 0.019 | 0.004 | 3.7e-08 | 30.4 | -0.037 | 0.035 | 0.291 |
| rs11614957 | A | G | 0.451 | 0.012 | 0.001 | 2.8e-30 | 131.9 | -0.016 | 0.012 | 0.199 |
| rs116173579 | T | C | 0.035 | -0.016 | 0.003 | 1.7e-08 | 31.7 | 0.058 | 0.038 | 0.133 |
| rs11633934 | A | C | 0.550 | 0.008 | 0.001 | 2.1e-14 | 58.5 | 0.009 | 0.012 | 0.481 |
| rs11643516 | T | C | 0.432 | -0.008 | 0.001 | 2.2e-14 | 58.7 | 0.001 | 0.012 | 0.917 |
| rs11664320 | T | C | 0.591 | 0.018 | 0.001 | 2.2e-62 | 275.4 | -0.012 | 0.013 | 0.337 |
| rs1167796 | A | G | 0.424 | 0.012 | 0.001 | 7.1e-30 | 128.8 | -0.009 | 0.012 | 0.483 |
| rs117005905 | T | C | 0.115 | 0.016 | 0.002 | 5.6e-22 | 92.5 | -0.017 | 0.020 | 0.403 |
| rs11724690 | T | G | 0.283 | 0.009 | 0.001 | 1.5e-16 | 68.1 | -0.007 | 0.013 | 0.587 |
| rs11746390 | T | C | 0.742 | -0.009 | 0.001 | 3.3e-13 | 53.1 | -0.009 | 0.014 | 0.526 |
| rs117494884 | T | C | 0.055 | -0.015 | 0.002 | 1.0e-10 | 41.9 | 0.050 | 0.033 | 0.133 |
| rs117520198 | A | G | 0.175 | 0.011 | 0.001 | 1.0e-15 | 64.7 | 0.001 | 0.017 | 0.964 |
| rs117520996 | T | G | 0.042 | 0.022 | 0.002 | 1.5e-18 | 77.2 | -0.013 | 0.026 | 0.626 |
| rs11757278 | T | C | 0.693 | -0.008 | 0.001 | 5.0e-13 | 52.7 | -0.001 | 0.013 | 0.958 |
| rs11772108 | A | T | 0.717 | -0.008 | 0.001 | 2.4e-12 | 49.1 | 0.010 | 0.013 | 0.465 |
| rs11773992 | C | G | 0.162 | -0.014 | 0.001 | 5.8e-24 | 101.5 | -0.016 | 0.019 | 0.401 |
| rs11774212 | T | C | 0.496 | 0.016 | 0.001 | 1.7e-50 | 222.7 | -0.007 | 0.012 | 0.600 |
| rs118134876 | T | C | 0.053 | -0.031 | 0.002 | 7.0e-46 | 201.5 | -0.032 | 0.034 | 0.350 |
| rs1182532 | A | C | 0.146 | 0.011 | 0.001 | 4.0e-14 | 57.5 | 0.025 | 0.017 | 0.155 |
| rs11861256 | A | C | 0.192 | 0.010 | 0.001 | 4.7e-14 | 57.0 | 0.009 | 0.015 | 0.556 |
| rs11925699 | A | G | 0.463 | -0.008 | 0.001 | 2.7e-13 | 53.2 | -0.012 | 0.012 | 0.343 |
| rs1193240 | A | C | 0.423 | 0.008 | 0.001 | 1.7e-15 | 63.8 | -0.001 | 0.012 | 0.915 |
| rs11942352 | A | G | 0.814 | -0.009 | 0.001 | 1.4e-11 | 45.9 | -0.006 | 0.015 | 0.680 |
| rs12055782 | A | G | 0.725 | -0.012 | 0.001 | 4.2e-24 | 103.1 | 0.012 | 0.013 | 0.357 |
| rs12089815 | A | G | 0.526 | 0.019 | 0.001 | 1.1e-69 | 311.4 | 0.006 | 0.013 | 0.665 |
| rs12113634 | T | C | 0.650 | 0.009 | 0.001 | 9.2e-17 | 69.4 | 0.037 | 0.014 | 0.007 |
| rs12126231 | A | G | 0.616 | 0.012 | 0.001 | 9.7e-28 | 118.2 | 0.001 | 0.013 | 0.918 |
| rs12142570 | A | T | 0.819 | 0.010 | 0.001 | 1.1e-12 | 50.9 | -0.004 | 0.019 | 0.834 |
| rs12150824 | A | G | 0.255 | 0.007 | 0.001 | 1.6e-09 | 36.4 | 0.007 | 0.014 | 0.609 |
| rs12151015 | C | G | 0.888 | -0.010 | 0.002 | 5.6e-09 | 34.0 | 0.029 | 0.018 | 0.117 |
| rs12155540 | T | C | 0.330 | 0.012 | 0.001 | 6.8e-26 | 111.5 | -0.002 | 0.013 | 0.875 |
| rs12201073 | T | C | 0.353 | -0.007 | 0.001 | 5.0e-11 | 43.0 | -0.013 | 0.013 | 0.325 |
| rs12234936 | T | C | 0.420 | -0.015 | 0.001 | 6.5e-43 | 189.7 | 0.017 | 0.013 | 0.192 |
| rs12240387 | A | G | 0.356 | -0.006 | 0.001 | 3.6e-08 | 30.2 | 0.004 | 0.013 | 0.757 |
| rs12273435 | A | G | 0.188 | -0.014 | 0.001 | 6.1e-27 | 115.7 | 0.004 | 0.016 | 0.796 |
| rs12339610 | T | C | 0.746 | 0.009 | 0.001 | 4.1e-14 | 56.8 | -0.002 | 0.013 | 0.883 |
| rs12362273 | A | C | 0.772 | -0.007 | 0.001 | 7.9e-09 | 33.1 | -0.021 | 0.015 | 0.159 |
| rs12431579 | A | G | 0.640 | -0.006 | 0.001 | 2.2e-08 | 31.2 | -0.005 | 0.013 | 0.724 |
| rs12435895 | A | G | 0.774 | -0.008 | 0.001 | 1.5e-10 | 40.8 | -0.019 | 0.014 | 0.163 |
| rs12506222 | T | C | 0.467 | -0.014 | 0.001 | 1.5e-41 | 183.7 | -0.015 | 0.012 | 0.225 |
| rs12524795 | T | C | 0.438 | 0.009 | 0.001 | 6.3e-16 | 65.7 | 0.013 | 0.013 | 0.293 |
| rs12569163 | T | G | 0.800 | -0.012 | 0.002 | 1.7e-11 | 45.4 | -0.014 | 0.017 | 0.392 |
| rs12609965 | A | C | 0.246 | 0.007 | 0.001 | 8.0e-09 | 33.5 | 0.009 | 0.014 | 0.488 |
| rs12632133 | A | G | 0.078 | 0.011 | 0.002 | 1.6e-08 | 31.9 | 0.000 | 0.022 | 0.993 |
| rs12699131 | A | G | 0.471 | 0.014 | 0.001 | 1.0e-39 | 173.2 | -0.017 | 0.012 | 0.165 |
| rs12709690 | T | C | 0.373 | -0.019 | 0.001 | 9.3e-65 | 290.6 | -0.009 | 0.014 | 0.503 |
| rs12764593 | C | G | 0.067 | 0.022 | 0.002 | 1.7e-23 | 100.1 | -0.016 | 0.018 | 0.393 |
| rs12768641 | A | G | 0.767 | 0.009 | 0.001 | 2.0e-14 | 58.4 | -0.017 | 0.014 | 0.232 |
| rs1278537 | T | G | 0.478 | -0.007 | 0.001 | 1.2e-10 | 41.2 | 0.013 | 0.013 | 0.340 |
| rs12828220 | T | C | 0.598 | -0.010 | 0.001 | 2.8e-19 | 80.0 | -0.017 | 0.013 | 0.184 |
| rs12893970 | A | G | 0.087 | -0.013 | 0.002 | 4.8e-13 | 52.2 | -0.011 | 0.025 | 0.649 |
| rs12897763 | A | G | 0.189 | 0.020 | 0.001 | 1.7e-49 | 219.0 | 0.020 | 0.015 | 0.164 |
| rs12915175 | A | G | 0.190 | -0.008 | 0.001 | 7.8e-10 | 38.0 | 0.021 | 0.015 | 0.164 |
| rs1291865 | T | G | 0.538 | -0.010 | 0.001 | 6.8e-23 | 96.4 | -0.016 | 0.013 | 0.193 |
| rs12937411 | T | C | 0.401 | 0.011 | 0.001 | 1.7e-23 | 100.8 | -0.022 | 0.013 | 0.080 |
| rs12962980 | C | G | 0.188 | 0.017 | 0.001 | 2.4e-35 | 153.0 | -0.012 | 0.017 | 0.475 |
| rs12974657 | T | C | 0.677 | 0.009 | 0.001 | 3.7e-16 | 65.9 | 0.021 | 0.013 | 0.115 |
| rs12981405 | T | C | 0.159 | -0.011 | 0.001 | 4.1e-15 | 61.5 | -0.004 | 0.018 | 0.823 |
| rs13010288 | T | G | 0.123 | 0.020 | 0.002 | 1.3e-37 | 163.9 | 0.009 | 0.021 | 0.679 |
| rs13030436 | T | C | 0.409 | 0.008 | 0.001 | 8.8e-14 | 55.1 | -0.031 | 0.013 | 0.015 |
| rs13122283 | A | G | 0.403 | 0.007 | 0.001 | 5.1e-11 | 43.0 | -0.008 | 0.013 | 0.529 |
| rs13133213 | A | G | 0.502 | 0.009 | 0.001 | 7.2e-17 | 69.1 | 0.014 | 0.012 | 0.269 |
| rs13135092 | A | G | 0.927 | 0.030 | 0.002 | 4.4e-58 | 258.4 | -0.093 | 0.047 | 0.048 |
| rs13138842 | A | G | 0.193 | 0.008 | 0.001 | 2.7e-10 | 39.9 | 0.022 | 0.016 | 0.159 |
| rs13159124 | A | G | 0.692 | -0.011 | 0.001 | 1.0e-23 | 101.3 | 0.000 | 0.013 | 0.986 |
| rs13161488 | T | C | 0.660 | -0.009 | 0.001 | 2.4e-15 | 62.4 | 0.015 | 0.013 | 0.236 |
| rs13212041 | T | C | 0.800 | 0.011 | 0.001 | 9.1e-18 | 73.5 | 0.028 | 0.015 | 0.074 |
| rs13214394 | A | T | 0.767 | -0.007 | 0.001 | 2.8e-08 | 31.0 | -0.019 | 0.016 | 0.223 |
| rs13244634 | T | C | 0.239 | -0.008 | 0.001 | 2.1e-10 | 40.6 | 0.005 | 0.014 | 0.750 |
| rs13252156 | A | C | 0.417 | 0.009 | 0.001 | 4.7e-17 | 70.6 | -0.018 | 0.013 | 0.177 |
| rs13278836 | A | G | 0.111 | 0.014 | 0.002 | 5.1e-17 | 70.1 | -0.008 | 0.018 | 0.654 |
| rs13284474 | T | C | 0.428 | 0.006 | 0.001 | 1.7e-09 | 36.6 | 0.004 | 0.013 | 0.748 |
| rs1329125 | T | C | 0.308 | -0.012 | 0.001 | 1.1e-28 | 123.2 | 0.023 | 0.013 | 0.087 |
| rs13332638 | A | G | 0.092 | -0.013 | 0.002 | 3.1e-12 | 48.6 | -0.009 | 0.019 | 0.621 |
| rs1334297 | A | G | 0.751 | 0.024 | 0.001 | 8.1e-90 | 406.8 | -0.004 | 0.014 | 0.768 |
| rs1337731 | A | G | 0.340 | 0.009 | 0.001 | 7.1e-15 | 60.4 | -0.016 | 0.014 | 0.263 |
| rs137121 | T | C | 0.127 | 0.013 | 0.002 | 8.1e-16 | 65.0 | -0.009 | 0.021 | 0.682 |
| rs1378893 | A | G | 0.226 | 0.009 | 0.001 | 1.5e-13 | 54.5 | -0.004 | 0.018 | 0.819 |
| rs1387148 | A | C | 0.193 | -0.009 | 0.001 | 5.9e-13 | 52.1 | 0.002 | 0.016 | 0.882 |
| rs1392816 | T | C | 0.372 | 0.010 | 0.001 | 5.4e-19 | 78.8 | -0.010 | 0.013 | 0.462 |
| rs139872934 | T | C | 0.094 | 0.014 | 0.002 | 3.2e-14 | 57.5 | 0.009 | 0.015 | 0.548 |
| rs1407350 | T | G | 0.647 | 0.008 | 0.001 | 9.1e-13 | 50.8 | -0.006 | 0.013 | 0.648 |
| rs141503359 | T | C | 0.014 | -0.028 | 0.005 | 3.0e-09 | 35.2 | 0.035 | 0.034 | 0.300 |
| rs14184 | C | G | 0.703 | -0.013 | 0.001 | 7.0e-32 | 138.8 | 0.001 | 0.013 | 0.949 |
| rs1452366 | T | G | 0.353 | -0.012 | 0.001 | 1.6e-27 | 117.2 | -0.008 | 0.013 | 0.566 |
| rs145481578 | T | C | 0.019 | 0.024 | 0.004 | 1.1e-09 | 37.2 | -0.039 | 0.053 | 0.457 |
| rs1464018 | T | C | 0.245 | -0.014 | 0.001 | 2.8e-26 | 113.1 | 0.002 | 0.013 | 0.863 |
| rs1483148 | T | G | 0.719 | 0.012 | 0.001 | 1.3e-24 | 105.0 | 0.012 | 0.013 | 0.366 |
| rs1483814 | T | G | 0.191 | 0.007 | 0.001 | 2.0e-08 | 31.4 | 0.028 | 0.016 | 0.074 |
| rs1485272 | T | C | 0.645 | -0.007 | 0.001 | 7.2e-11 | 42.6 | -0.008 | 0.013 | 0.521 |
| rs1490834 | A | G | 0.550 | -0.006 | 0.001 | 1.1e-09 | 37.0 | 0.001 | 0.013 | 0.950 |
| rs149285517 | T | C | 0.123 | -0.012 | 0.002 | 4.6e-13 | 52.4 | -0.015 | 0.017 | 0.383 |
| rs150121450 | T | G | 0.029 | 0.020 | 0.003 | 1.0e-10 | 41.8 | -0.004 | 0.032 | 0.893 |
| rs1516036 | A | G | 0.547 | 0.007 | 0.001 | 1.6e-11 | 45.3 | -0.008 | 0.013 | 0.519 |
| rs1585524 | A | G | 0.473 | 0.006 | 0.001 | 4.7e-08 | 29.5 | -0.003 | 0.013 | 0.837 |
| rs158839 | A | C | 0.617 | -0.006 | 0.001 | 6.4e-09 | 33.9 | -0.012 | 0.013 | 0.358 |
| rs1632822 | C | G | 0.369 | 0.006 | 0.001 | 4.9e-08 | 29.6 | 0.010 | 0.013 | 0.431 |
| rs165633 | A | G | 0.784 | -0.012 | 0.001 | 2.2e-23 | 98.9 | -0.005 | 0.013 | 0.673 |
| rs16844417 | A | G | 0.109 | -0.009 | 0.002 | 1.1e-08 | 32.7 | -0.004 | 0.018 | 0.847 |
| rs1689510 | C | G | 0.314 | 0.017 | 0.001 | 4.0e-55 | 242.5 | 0.026 | 0.013 | 0.047 |
| rs16919774 | A | T | 0.795 | -0.009 | 0.001 | 2.7e-13 | 53.4 | 0.006 | 0.014 | 0.634 |
| rs16962221 | A | G | 0.351 | 0.007 | 0.001 | 1.1e-11 | 46.2 | 0.009 | 0.013 | 0.500 |
| rs16983844 | A | G | 0.188 | 0.011 | 0.001 | 7.9e-17 | 69.3 | -0.028 | 0.016 | 0.085 |
| rs17088142 | T | G | 0.453 | -0.007 | 0.001 | 7.5e-11 | 42.3 | -0.019 | 0.013 | 0.134 |
| rs17096452 | A | T | 0.791 | 0.011 | 0.001 | 3.2e-19 | 80.3 | 0.007 | 0.016 | 0.651 |
| rs17126938 | T | C | 0.858 | -0.013 | 0.002 | 5.8e-17 | 70.1 | -0.001 | 0.022 | 0.974 |
| rs171697 | C | G | 0.688 | 0.015 | 0.001 | 2.4e-42 | 186.8 | -0.015 | 0.014 | 0.262 |
| rs17224289 | T | C | 0.118 | -0.013 | 0.002 | 3.1e-16 | 66.9 | 0.048 | 0.019 | 0.014 |
| rs17248751 | A | G | 0.799 | -0.014 | 0.001 | 3.4e-27 | 117.2 | -0.049 | 0.018 | 0.005 |
| rs17257579 | T | C | 0.879 | -0.013 | 0.002 | 1.4e-16 | 67.9 | -0.049 | 0.022 | 0.028 |
| rs17266097 | T | C | 0.386 | 0.009 | 0.001 | 2.0e-16 | 67.5 | -0.020 | 0.013 | 0.118 |
| rs1738050 | C | G | 0.630 | -0.011 | 0.001 | 1.5e-24 | 103.7 | -0.002 | 0.013 | 0.863 |
| rs17503473 | T | C | 0.705 | 0.012 | 0.001 | 1.1e-25 | 109.7 | 0.009 | 0.013 | 0.482 |
| rs17563464 | A | C | 0.203 | -0.013 | 0.001 | 2.4e-23 | 99.7 | 0.011 | 0.018 | 0.540 |
| rs17565975 | A | G | 0.530 | -0.012 | 0.001 | 1.2e-30 | 133.0 | -0.003 | 0.012 | 0.786 |
| rs17649841 | A | C | 0.227 | -0.008 | 0.001 | 8.4e-11 | 42.3 | -0.019 | 0.014 | 0.156 |
| rs17669337 | T | C | 0.405 | -0.010 | 0.001 | 2.5e-21 | 90.4 | -0.012 | 0.013 | 0.357 |
| rs17721326 | T | C | 0.292 | 0.009 | 0.001 | 4.8e-14 | 56.6 | 0.022 | 0.013 | 0.109 |
| rs17812330 | T | G | 0.257 | -0.008 | 0.001 | 8.9e-12 | 46.8 | -0.013 | 0.013 | 0.333 |
| rs1786313 | T | C | 0.309 | 0.008 | 0.001 | 8.1e-12 | 46.6 | 0.019 | 0.013 | 0.160 |
| rs17881016 | A | G | 0.742 | -0.013 | 0.001 | 5.3e-28 | 119.9 | 0.013 | 0.014 | 0.350 |
| rs181164682 | A | T | 0.015 | 0.024 | 0.004 | 8.2e-11 | 42.2 | 0.023 | 0.061 | 0.709 |
| rs1820986 | A | G | 0.147 | -0.012 | 0.002 | 2.3e-14 | 58.2 | -0.028 | 0.020 | 0.170 |
| rs1835388 | T | C | 0.483 | -0.008 | 0.001 | 7.7e-15 | 60.2 | -0.008 | 0.012 | 0.516 |
| rs184654 | C | G | 0.838 | 0.016 | 0.001 | 1.1e-30 | 133.1 | 0.022 | 0.014 | 0.118 |
| rs1861786 | A | G | 0.377 | -0.008 | 0.001 | 1.3e-12 | 50.0 | 0.013 | 0.013 | 0.300 |
| rs1865880 | T | C | 0.355 | -0.006 | 0.001 | 1.6e-08 | 31.8 | 0.015 | 0.013 | 0.260 |
| rs1866710 | A | G | 0.286 | -0.014 | 0.001 | 1.1e-35 | 156.0 | 0.046 | 0.015 | 0.003 |
| rs1866823 | A | G | 0.542 | 0.011 | 0.001 | 8.1e-24 | 101.5 | -0.015 | 0.013 | 0.238 |
| rs1871745 | A | G | 0.766 | 0.010 | 0.001 | 2.2e-15 | 62.4 | -0.015 | 0.014 | 0.289 |
| rs1880692 | A | G | 0.542 | 0.008 | 0.001 | 2.8e-13 | 53.4 | 0.016 | 0.012 | 0.210 |
| rs1884368 | T | C | 0.232 | 0.009 | 0.001 | 7.7e-14 | 55.5 | -0.025 | 0.015 | 0.099 |
| rs1888765 | C | G | 0.767 | 0.009 | 0.001 | 7.1e-13 | 51.1 | -0.027 | 0.015 | 0.067 |
| rs192230289 | T | C | 0.016 | -0.027 | 0.004 | 5.2e-11 | 43.1 | 0.021 | 0.043 | 0.626 |
| rs192436652 | T | C | 0.027 | -0.028 | 0.003 | 9.9e-17 | 68.9 | 0.037 | 0.029 | 0.191 |
| rs1971218 | A | G | 0.519 | 0.009 | 0.001 | 5.8e-16 | 66.0 | 0.018 | 0.012 | 0.153 |
| rs1980099 | A | G | 0.796 | 0.011 | 0.001 | 2.6e-16 | 67.4 | 0.001 | 0.015 | 0.927 |
| rs2006853 | A | G | 0.576 | 0.009 | 0.001 | 4.0e-18 | 74.7 | 0.008 | 0.013 | 0.554 |
| rs2026037 | T | C | 0.849 | 0.015 | 0.001 | 4.6e-27 | 116.7 | -0.017 | 0.016 | 0.281 |
| rs2055940 | A | G | 0.307 | 0.007 | 0.001 | 3.3e-10 | 39.4 | 0.005 | 0.016 | 0.759 |
| rs2063569 | C | G | 0.374 | -0.010 | 0.001 | 6.1e-19 | 79.3 | -0.009 | 0.014 | 0.494 |
| rs211283 | C | G | 0.233 | -0.009 | 0.001 | 5.2e-14 | 56.2 | 0.009 | 0.016 | 0.586 |
| rs213014 | T | C | 0.548 | -0.007 | 0.001 | 1.5e-10 | 41.1 | -0.005 | 0.012 | 0.664 |
| rs2154209 | T | C | 0.801 | -0.008 | 0.001 | 2.5e-09 | 35.4 | 0.018 | 0.015 | 0.246 |
| rs2160514 | A | C | 0.568 | -0.011 | 0.001 | 5.9e-26 | 112.0 | -0.010 | 0.013 | 0.430 |
| rs2174752 | T | G | 0.435 | -0.009 | 0.001 | 2.4e-16 | 67.4 | 0.018 | 0.012 | 0.158 |
| rs2177083 | A | G | 0.502 | -0.007 | 0.001 | 1.5e-12 | 49.8 | -0.005 | 0.013 | 0.708 |
| rs2195041 | A | G | 0.393 | 0.007 | 0.001 | 6.2e-10 | 37.9 | -0.006 | 0.012 | 0.616 |
| rs2210174 | T | C | 0.622 | 0.006 | 0.001 | 1.3e-08 | 32.2 | 0.008 | 0.013 | 0.499 |
| rs2214631 | A | T | 0.311 | 0.007 | 0.001 | 1.4e-09 | 36.4 | 0.016 | 0.014 | 0.257 |
| rs2216053 | T | G | 0.737 | -0.007 | 0.001 | 1.1e-09 | 37.3 | 0.016 | 0.014 | 0.231 |
| rs2222760 | A | G | 0.272 | -0.008 | 0.001 | 8.2e-12 | 46.9 | 0.003 | 0.015 | 0.838 |
| rs2238483 | A | G | 0.815 | 0.010 | 0.001 | 5.3e-13 | 51.9 | -0.006 | 0.016 | 0.706 |
| rs2239736 | T | C | 0.510 | 0.009 | 0.001 | 3.0e-16 | 66.5 | -0.004 | 0.013 | 0.750 |
| rs2275154 | A | G | 0.675 | 0.016 | 0.001 | 1.7e-44 | 197.0 | 0.023 | 0.014 | 0.094 |
| rs2289769 | A | G | 0.869 | -0.008 | 0.002 | 2.8e-08 | 30.8 | 0.022 | 0.019 | 0.257 |
| rs2297293 | C | G | 0.326 | 0.008 | 0.001 | 2.3e-12 | 49.0 | -0.017 | 0.013 | 0.180 |
| rs2301015 | T | C | 0.341 | -0.011 | 0.001 | 1.4e-21 | 90.4 | 0.005 | 0.014 | 0.706 |
| rs2303907 | T | C | 0.518 | 0.009 | 0.001 | 4.2e-16 | 65.8 | -0.013 | 0.012 | 0.304 |
| rs2304282 | A | G | 0.407 | 0.011 | 0.001 | 4.0e-25 | 107.7 | -0.028 | 0.013 | 0.027 |
| rs2352430 | T | C | 0.770 | -0.008 | 0.001 | 3.1e-10 | 39.9 | 0.000 | 0.016 | 0.976 |
| rs2364972 | A | G | 0.548 | 0.007 | 0.001 | 7.7e-12 | 46.9 | 0.021 | 0.013 | 0.096 |
| rs2371001 | A | G | 0.501 | -0.009 | 0.001 | 7.2e-19 | 78.3 | -0.017 | 0.012 | 0.172 |
| rs2430813 | T | C | 0.765 | 0.011 | 0.001 | 8.6e-19 | 77.9 | -0.013 | 0.015 | 0.379 |
| rs2447091 | T | C | 0.627 | 0.009 | 0.001 | 2.4e-17 | 71.3 | 0.021 | 0.013 | 0.096 |
| rs2498018 | C | G | 0.186 | -0.009 | 0.001 | 1.9e-11 | 45.1 | -0.030 | 0.016 | 0.056 |
| rs2504846 | T | C | 0.032 | -0.026 | 0.003 | 3.7e-15 | 61.8 | -0.068 | 0.033 | 0.039 |
| rs252991 | A | G | 0.369 | 0.010 | 0.001 | 2.3e-21 | 90.2 | 0.005 | 0.014 | 0.716 |
| rs2535692 | A | G | 0.282 | 0.007 | 0.001 | 5.2e-09 | 34.4 | -0.005 | 0.014 | 0.715 |
| rs2570492 | A | G | 0.396 | 0.013 | 0.001 | 5.9e-34 | 147.4 | 0.021 | 0.013 | 0.096 |
| rs2610990 | A | G | 0.274 | 0.012 | 0.001 | 3.5e-22 | 93.1 | -0.011 | 0.013 | 0.391 |
| rs2624841 | T | C | 0.330 | -0.026 | 0.001 | 2.6e-126 | 567.8 | -0.012 | 0.014 | 0.402 |
| rs2630767 | T | C | 0.474 | 0.013 | 0.001 | 6.6e-35 | 153.3 | 0.008 | 0.012 | 0.514 |
| rs2631535 | A | G | 0.336 | 0.012 | 0.001 | 2.4e-28 | 122.8 | 0.015 | 0.013 | 0.244 |
| rs2639655 | C | G | 0.806 | 0.009 | 0.001 | 3.5e-12 | 48.1 | 0.028 | 0.016 | 0.074 |
| rs2668196 | A | T | 0.208 | -0.012 | 0.001 | 3.3e-19 | 80.7 | -0.027 | 0.018 | 0.146 |
| rs273028 | A | T | 0.086 | -0.014 | 0.002 | 9.3e-12 | 46.2 | -0.016 | 0.028 | 0.558 |
| rs2764684 | T | C | 0.847 | 0.015 | 0.001 | 2.6e-27 | 116.6 | 0.003 | 0.020 | 0.882 |
| rs28381527 | A | G | 0.077 | -0.020 | 0.002 | 9.7e-25 | 105.1 | 0.001 | 0.020 | 0.957 |
| rs2841425 | A | C | 0.180 | -0.009 | 0.001 | 4.4e-10 | 38.8 | -0.002 | 0.015 | 0.907 |
| rs28438078 | A | G | 0.142 | -0.010 | 0.001 | 1.7e-11 | 45.4 | 0.015 | 0.019 | 0.423 |
| rs28458909 | T | C | 0.126 | -0.019 | 0.002 | 6.0e-31 | 134.5 | 0.012 | 0.016 | 0.437 |
| rs28588750 | A | G | 0.655 | 0.011 | 0.001 | 7.6e-26 | 110.5 | 0.022 | 0.013 | 0.104 |
| rs28617748 | A | T | 0.997 | 0.009 | 0.001 | 1.1e-12 | 50.4 | -0.008 | 0.013 | 0.539 |
| rs28807201 | T | C | 0.684 | -0.011 | 0.001 | 2.0e-23 | 99.8 | 0.000 | 0.014 | 0.995 |
| rs2884364 | A | G | 0.374 | -0.016 | 0.001 | 1.3e-49 | 218.1 | 0.001 | 0.013 | 0.944 |
| rs2901785 | A | G | 0.469 | 0.014 | 0.001 | 4.5e-41 | 180.8 | -0.012 | 0.012 | 0.348 |
| rs2903624 | A | G | 0.468 | -0.006 | 0.001 | 1.2e-08 | 32.5 | 0.020 | 0.012 | 0.112 |
| rs2916490 | A | G | 0.305 | -0.010 | 0.001 | 4.0e-20 | 85.0 | -0.005 | 0.015 | 0.734 |
| rs2964252 | A | G | 0.322 | 0.010 | 0.001 | 1.3e-19 | 82.1 | 0.003 | 0.013 | 0.828 |
| rs2974337 | T | C | 0.478 | 0.012 | 0.001 | 2.4e-29 | 126.3 | 0.002 | 0.012 | 0.861 |
| rs3013170 | T | C | 0.596 | 0.007 | 0.001 | 4.5e-11 | 43.7 | 0.015 | 0.013 | 0.264 |
| rs303752 | A | G | 0.402 | -0.006 | 0.001 | 1.5e-08 | 31.9 | -0.026 | 0.013 | 0.037 |
| rs3095075 | A | G | 0.550 | -0.014 | 0.001 | 3.8e-38 | 167.8 | 0.004 | 0.013 | 0.753 |
| rs3098650 | C | G | 0.602 | 0.012 | 0.001 | 7.7e-28 | 119.6 | -0.003 | 0.013 | 0.789 |
| rs3108680 | T | C | 0.668 | 0.010 | 0.001 | 1.9e-20 | 86.0 | 0.001 | 0.014 | 0.914 |
| rs329120 | T | C | 0.425 | 0.013 | 0.001 | 5.2e-36 | 156.7 | -0.015 | 0.013 | 0.231 |
| rs336433 | A | G | 0.422 | 0.008 | 0.001 | 2.1e-13 | 54.5 | 0.000 | 0.013 | 0.995 |
| rs34305371 | A | G | 0.086 | 0.031 | 0.002 | 1.8e-71 | 318.9 | 0.047 | 0.021 | 0.025 |
| rs34316 | A | C | 0.435 | 0.016 | 0.001 | 6.6e-53 | 236.2 | 0.002 | 0.013 | 0.896 |
| rs34488670 | T | C | 0.786 | 0.014 | 0.001 | 6.8e-28 | 120.0 | 0.011 | 0.015 | 0.473 |
| rs34719425 | T | C | 0.251 | 0.010 | 0.001 | 8.1e-15 | 60.8 | 0.003 | 0.014 | 0.825 |
| rs34743418 | T | C | 0.464 | -0.010 | 0.001 | 5.0e-21 | 88.8 | -0.008 | 0.012 | 0.503 |
| rs34984805 | T | C | 0.530 | 0.007 | 0.001 | 1.5e-12 | 49.7 | 0.010 | 0.012 | 0.418 |
| rs35226705 | A | C | 0.544 | 0.006 | 0.001 | 4.9e-09 | 34.2 | -0.009 | 0.012 | 0.466 |
| rs35319653 | T | C | 0.326 | 0.015 | 0.001 | 2.2e-40 | 178.6 | 0.016 | 0.013 | 0.232 |
| rs35375125 | T | C | 0.622 | -0.009 | 0.001 | 5.2e-18 | 75.3 | 0.020 | 0.013 | 0.105 |
| rs35414043 | A | G | 0.077 | -0.015 | 0.002 | 5.6e-15 | 61.0 | -0.039 | 0.023 | 0.085 |
| rs35414759 | T | C | 0.830 | -0.011 | 0.001 | 4.7e-16 | 65.7 | -0.027 | 0.016 | 0.082 |
| rs35565533 | A | C | 0.744 | 0.008 | 0.001 | 1.2e-12 | 50.1 | 0.029 | 0.017 | 0.082 |
| rs35583726 | A | G | 0.836 | 0.012 | 0.001 | 3.1e-17 | 71.6 | -0.001 | 0.019 | 0.977 |
| rs356903 | T | C | 0.267 | -0.007 | 0.001 | 2.5e-09 | 35.3 | 0.010 | 0.015 | 0.497 |
| rs35733856 | A | G | 0.560 | -0.012 | 0.001 | 8.3e-29 | 123.3 | -0.015 | 0.012 | 0.241 |
| rs35951883 | A | G | 0.790 | -0.008 | 0.001 | 2.3e-10 | 40.2 | 0.015 | 0.018 | 0.394 |
| rs36048136 | A | G | 0.208 | 0.012 | 0.001 | 4.7e-21 | 89.0 | -0.004 | 0.015 | 0.773 |
| rs3731507 | T | C | 0.052 | 0.028 | 0.003 | 1.7e-26 | 113.4 | 0.017 | 0.018 | 0.349 |
| rs3747631 | C | G | 0.201 | 0.020 | 0.001 | 7.2e-58 | 258.8 | 0.020 | 0.015 | 0.178 |
| rs3783006 | C | G | 0.419 | 0.013 | 0.001 | 1.7e-35 | 155.4 | -0.007 | 0.013 | 0.566 |
| rs378342 | T | C | 0.270 | -0.008 | 0.001 | 5.4e-12 | 47.7 | 0.007 | 0.014 | 0.605 |
| rs3796432 | T | G | 0.366 | 0.007 | 0.001 | 2.7e-10 | 40.1 | -0.015 | 0.013 | 0.260 |
| rs382196 | T | G | 0.361 | -0.007 | 0.001 | 7.5e-11 | 42.4 | -0.005 | 0.013 | 0.674 |
| rs3863241 | T | C | 0.536 | -0.009 | 0.001 | 2.9e-16 | 66.9 | 0.013 | 0.013 | 0.300 |
| rs3869097 | T | C | 0.333 | -0.009 | 0.001 | 1.1e-15 | 64.6 | 0.003 | 0.013 | 0.807 |
| rs4076457 | T | C | 0.238 | 0.012 | 0.001 | 4.5e-24 | 102.0 | -0.026 | 0.015 | 0.082 |
| rs4127499 | A | G | 0.325 | 0.011 | 0.001 | 2.9e-23 | 99.3 | 0.009 | 0.014 | 0.516 |
| rs4141463 | T | C | 0.429 | -0.009 | 0.001 | 3.9e-16 | 66.4 | -0.029 | 0.013 | 0.025 |
| rs41741 | T | G | 0.663 | 0.007 | 0.001 | 3.4e-11 | 44.1 | 0.024 | 0.013 | 0.063 |
| rs421154 | C | G | 0.781 | 0.011 | 0.001 | 2.1e-18 | 77.2 | 0.009 | 0.017 | 0.604 |
| rs42210 | C | G | 0.692 | -0.012 | 0.001 | 3.1e-24 | 103.0 | 0.005 | 0.013 | 0.685 |
| rs422247 | T | C | 0.739 | 0.008 | 0.001 | 3.6e-10 | 39.4 | 0.002 | 0.015 | 0.911 |
| rs424387 | C | G | 0.599 | -0.006 | 0.001 | 7.1e-09 | 33.3 | -0.004 | 0.013 | 0.774 |
| rs4279337 | A | T | 0.258 | 0.009 | 0.001 | 4.2e-13 | 53.0 | -0.010 | 0.015 | 0.499 |
| rs4358081 | A | C | 0.534 | -0.010 | 0.001 | 2.1e-20 | 85.3 | -0.017 | 0.012 | 0.179 |
| rs4375600 | A | G | 0.105 | -0.010 | 0.002 | 3.2e-08 | 30.6 | 0.005 | 0.017 | 0.787 |
| rs4390219 | T | C | 0.314 | -0.007 | 0.001 | 3.4e-11 | 43.8 | -0.014 | 0.013 | 0.291 |
| rs4391653 | T | C | 0.254 | -0.008 | 0.001 | 6.9e-12 | 47.0 | 0.010 | 0.014 | 0.485 |
| rs4409028 | A | C | 0.636 | 0.006 | 0.001 | 4.9e-08 | 29.9 | 0.008 | 0.013 | 0.518 |
| rs4447403 | T | C | 0.743 | 0.007 | 0.001 | 7.0e-09 | 33.5 | -0.002 | 0.014 | 0.887 |
| rs4467547 | T | G | 0.384 | 0.013 | 0.001 | 3.4e-35 | 152.6 | -0.019 | 0.013 | 0.148 |
| rs4551987 | A | G | 0.839 | 0.012 | 0.001 | 1.6e-18 | 77.2 | -0.005 | 0.014 | 0.722 |
| rs4557790 | A | C | 0.567 | 0.020 | 0.001 | 4.9e-84 | 380.8 | 0.002 | 0.012 | 0.885 |
| rs4583487 | T | G | 0.380 | 0.023 | 0.001 | 2.6e-102 | 461.2 | -0.009 | 0.013 | 0.495 |
| rs4588749 | A | G | 0.441 | 0.013 | 0.001 | 7.6e-33 | 143.1 | 0.009 | 0.012 | 0.485 |
| rs4650228 | A | G | 0.462 | -0.008 | 0.001 | 6.7e-16 | 65.1 | 0.000 | 0.013 | 0.985 |
| rs4663617 | A | T | 0.230 | 0.010 | 0.001 | 3.2e-17 | 70.8 | 0.004 | 0.017 | 0.831 |
| rs4667878 | A | T | 0.186 | 0.008 | 0.001 | 1.7e-08 | 31.9 | 0.006 | 0.015 | 0.706 |
| rs4679353 | A | G | 0.615 | 0.007 | 0.001 | 3.0e-11 | 44.1 | 0.011 | 0.013 | 0.418 |
| rs4685448 | A | G | 0.421 | -0.007 | 0.001 | 1.2e-10 | 41.8 | -0.023 | 0.012 | 0.065 |
| rs4726070 | A | G | 0.593 | 0.010 | 0.001 | 1.1e-21 | 91.1 | 0.006 | 0.012 | 0.636 |
| rs474000 | A | G | 0.430 | 0.006 | 0.001 | 1.1e-09 | 37.4 | 0.027 | 0.012 | 0.031 |
| rs4741600 | T | C | 0.345 | 0.016 | 0.001 | 2.1e-46 | 206.3 | -0.005 | 0.013 | 0.701 |
| rs4751360 | T | C | 0.469 | 0.009 | 0.001 | 4.0e-18 | 75.1 | -0.003 | 0.013 | 0.806 |
| rs4766975 | A | G | 0.615 | -0.009 | 0.001 | 4.4e-16 | 66.3 | -0.008 | 0.013 | 0.535 |
| rs4772268 | A | G | 0.333 | 0.010 | 0.001 | 4.0e-19 | 79.9 | 0.015 | 0.013 | 0.274 |
| rs4788080 | T | C | 0.356 | -0.013 | 0.001 | 1.2e-34 | 150.9 | -0.009 | 0.013 | 0.470 |
| rs4798783 | A | G | 0.705 | -0.012 | 0.001 | 1.3e-23 | 100.3 | -0.005 | 0.014 | 0.700 |
| rs4805761 | A | G | 0.150 | -0.014 | 0.001 | 6.0e-22 | 92.6 | 0.001 | 0.018 | 0.950 |
| rs4812818 | T | G | 0.184 | -0.007 | 0.001 | 2.2e-08 | 31.3 | 0.014 | 0.015 | 0.353 |
| rs4818226 | A | G | 0.314 | 0.011 | 0.001 | 1.5e-23 | 100.4 | 0.019 | 0.013 | 0.143 |
| rs4846724 | A | G | 0.532 | 0.008 | 0.001 | 2.6e-13 | 53.4 | 0.025 | 0.013 | 0.044 |
| rs4850810 | T | G | 0.520 | -0.013 | 0.001 | 6.7e-36 | 156.8 | 0.030 | 0.013 | 0.018 |
| rs4864881 | T | C | 0.596 | -0.007 | 0.001 | 7.7e-11 | 42.6 | -0.006 | 0.013 | 0.658 |
| rs4869579 | T | C | 0.516 | 0.006 | 0.001 | 1.4e-09 | 36.5 | 0.020 | 0.013 | 0.107 |
| rs4869852 | T | C | 0.671 | -0.008 | 0.001 | 1.5e-13 | 54.2 | -0.004 | 0.013 | 0.731 |
| rs4876775 | A | G | 0.338 | -0.011 | 0.001 | 1.6e-23 | 99.3 | -0.010 | 0.013 | 0.441 |
| rs4879832 | T | C | 0.391 | -0.006 | 0.001 | 3.6e-09 | 34.8 | -0.012 | 0.013 | 0.365 |
| rs4882141 | T | C | 0.649 | 0.007 | 0.001 | 1.5e-09 | 36.2 | -0.029 | 0.014 | 0.030 |
| rs4883624 | T | C | 0.466 | -0.007 | 0.001 | 6.0e-11 | 43.1 | 0.014 | 0.012 | 0.272 |
| rs489408 | A | G | 0.436 | 0.008 | 0.001 | 3.2e-13 | 53.2 | -0.002 | 0.012 | 0.868 |
| rs490535 | T | C | 0.338 | 0.007 | 0.001 | 3.8e-10 | 39.2 | 0.008 | 0.013 | 0.532 |
| rs4911257 | T | C | 0.607 | 0.010 | 0.001 | 8.0e-20 | 83.7 | 0.011 | 0.013 | 0.394 |
| rs4989480 | A | G | 0.857 | -0.009 | 0.002 | 4.0e-09 | 34.5 | 0.021 | 0.018 | 0.267 |
| rs5021426 | T | C | 0.716 | -0.011 | 0.001 | 1.7e-21 | 91.0 | 0.031 | 0.013 | 0.020 |
| rs55640000 | T | G | 0.634 | -0.008 | 0.001 | 1.4e-12 | 50.4 | 0.005 | 0.013 | 0.683 |
| rs55897719 | A | C | 0.287 | -0.013 | 0.001 | 2.0e-28 | 121.4 | 0.004 | 0.013 | 0.786 |
| rs55975662 | A | G | 0.846 | 0.015 | 0.001 | 1.1e-24 | 105.7 | 0.012 | 0.018 | 0.522 |
| rs56016333 | T | C | 0.651 | -0.014 | 0.001 | 4.4e-36 | 158.1 | 0.009 | 0.013 | 0.473 |
| rs56059718 | A | C | 0.197 | -0.012 | 0.001 | 3.8e-20 | 84.9 | 0.030 | 0.016 | 0.052 |
| rs56133711 | A | G | 0.224 | -0.012 | 0.001 | 7.2e-24 | 102.3 | 0.002 | 0.014 | 0.885 |
| rs561655 | A | G | 0.653 | -0.009 | 0.001 | 5.7e-16 | 65.6 | 0.018 | 0.013 | 0.154 |
| rs56188374 | A | G | 0.070 | -0.014 | 0.002 | 2.7e-13 | 53.4 | 0.011 | 0.019 | 0.575 |
| rs56194430 | T | C | 0.161 | -0.019 | 0.001 | 5.7e-40 | 174.2 | -0.014 | 0.019 | 0.446 |
| rs56306348 | A | G | 0.625 | 0.007 | 0.001 | 3.8e-12 | 48.0 | 0.018 | 0.014 | 0.175 |
| rs56355837 | A | C | 0.686 | -0.008 | 0.001 | 2.9e-14 | 57.5 | 0.008 | 0.013 | 0.540 |
| rs56378598 | T | C | 0.291 | 0.008 | 0.001 | 4.3e-12 | 48.2 | -0.003 | 0.013 | 0.786 |
| rs56409354 | A | G | 0.218 | -0.016 | 0.001 | 1.6e-36 | 158.5 | 0.012 | 0.015 | 0.434 |
| rs565960 | T | C | 0.702 | 0.009 | 0.001 | 3.8e-14 | 56.9 | 0.029 | 0.014 | 0.038 |
| rs567775 | T | C | 0.477 | 0.007 | 0.001 | 2.7e-11 | 44.1 | 0.029 | 0.012 | 0.021 |
| rs57349798 | A | G | 0.399 | 0.010 | 0.001 | 4.3e-19 | 80.3 | 0.008 | 0.013 | 0.560 |
| rs5759002 | A | G | 0.508 | 0.008 | 0.001 | 2.0e-15 | 62.8 | -0.002 | 0.012 | 0.870 |
| rs5765717 | T | G | 0.478 | -0.007 | 0.001 | 4.7e-12 | 47.5 | -0.002 | 0.012 | 0.844 |
| rs586829 | A | G | 0.634 | 0.010 | 0.001 | 5.5e-20 | 84.4 | -0.014 | 0.013 | 0.278 |
| rs58694847 | C | G | 0.276 | -0.012 | 0.001 | 7.8e-23 | 97.3 | -0.003 | 0.014 | 0.799 |
| rs590013 | T | C | 0.650 | 0.011 | 0.001 | 3.0e-23 | 99.5 | 0.014 | 0.013 | 0.310 |
| rs59903549 | T | C | 0.085 | -0.015 | 0.002 | 3.9e-14 | 57.5 | -0.055 | 0.030 | 0.069 |
| rs60053512 | A | G | 0.167 | 0.012 | 0.001 | 1.3e-17 | 73.3 | -0.004 | 0.014 | 0.784 |
| rs6020560 | T | C | 0.514 | -0.010 | 0.001 | 6.9e-20 | 83.1 | -0.016 | 0.012 | 0.210 |
| rs6028084 | T | C | 0.611 | 0.015 | 0.001 | 3.4e-45 | 200.2 | 0.021 | 0.013 | 0.102 |
| rs60307735 | T | C | 0.329 | 0.009 | 0.001 | 6.8e-17 | 69.1 | 0.019 | 0.013 | 0.125 |
| rs6044142 | A | G | 0.584 | -0.007 | 0.001 | 2.7e-12 | 48.6 | 0.008 | 0.013 | 0.517 |
| rs60814418 | T | C | 0.214 | -0.023 | 0.001 | 5.2e-72 | 322.6 | 0.068 | 0.022 | 0.002 |
| rs6088618 | A | G | 0.438 | 0.008 | 0.001 | 1.1e-14 | 60.0 | 0.003 | 0.012 | 0.785 |
| rs6091534 | T | C | 0.368 | 0.007 | 0.001 | 7.9e-12 | 46.7 | 0.014 | 0.013 | 0.285 |
| rs6093705 | A | C | 0.683 | 0.010 | 0.001 | 1.9e-17 | 72.6 | 0.011 | 0.015 | 0.446 |
| rs61160187 | A | G | 0.608 | -0.020 | 0.001 | 1.3e-74 | 336.6 | 0.010 | 0.013 | 0.447 |
| rs61747226 | T | C | 0.041 | -0.019 | 0.003 | 3.4e-13 | 52.8 | -0.081 | 0.044 | 0.067 |
| rs61964892 | T | C | 0.782 | -0.008 | 0.001 | 8.8e-10 | 37.6 | -0.004 | 0.014 | 0.801 |
| rs62079997 | T | C | 0.544 | -0.007 | 0.001 | 1.5e-10 | 40.8 | 0.006 | 0.012 | 0.609 |
| rs62086577 | A | G | 0.211 | 0.007 | 0.001 | 8.1e-09 | 33.3 | -0.028 | 0.015 | 0.072 |
| rs62114178 | A | G | 0.360 | 0.007 | 0.001 | 2.5e-10 | 40.2 | 0.000 | 0.015 | 0.983 |
| rs62156740 | A | T | 0.985 | -0.029 | 0.004 | 1.2e-13 | 55.2 | 0.000 | 0.051 | 0.993 |
| rs62172117 | A | G | 0.343 | 0.014 | 0.001 | 3.3e-35 | 152.0 | -0.006 | 0.013 | 0.643 |
| rs62199988 | C | G | 0.622 | -0.009 | 0.001 | 7.9e-16 | 64.9 | -0.001 | 0.013 | 0.928 |
| rs62244884 | A | G | 0.408 | 0.017 | 0.001 | 4.0e-55 | 243.8 | -0.014 | 0.013 | 0.259 |
| rs62247449 | C | G | 0.411 | 0.010 | 0.001 | 1.5e-19 | 81.2 | -0.003 | 0.013 | 0.820 |
| rs62262312 | C | G | 0.151 | 0.019 | 0.001 | 2.4e-40 | 177.0 | 0.020 | 0.018 | 0.271 |
| rs623200 | T | C | 0.398 | 0.007 | 0.001 | 5.8e-11 | 42.5 | 0.003 | 0.013 | 0.793 |
| rs62367470 | T | C | 0.184 | 0.008 | 0.001 | 5.8e-09 | 34.0 | -0.014 | 0.018 | 0.439 |
| rs62439683 | A | C | 0.244 | -0.012 | 0.001 | 1.5e-23 | 99.5 | -0.009 | 0.015 | 0.553 |
| rs62543169 | T | C | 0.404 | -0.012 | 0.001 | 3.3e-28 | 120.8 | -0.006 | 0.013 | 0.667 |
| rs6428152 | A | G | 0.290 | -0.008 | 0.001 | 5.5e-12 | 47.3 | -0.004 | 0.013 | 0.786 |
| rs6440854 | A | G | 0.872 | 0.009 | 0.002 | 2.6e-09 | 35.6 | 0.008 | 0.015 | 0.593 |
| rs6457796 | T | C | 0.713 | 0.010 | 0.001 | 4.8e-19 | 79.0 | -0.009 | 0.014 | 0.514 |
| rs6466819 | A | G | 0.371 | -0.010 | 0.001 | 2.5e-21 | 89.5 | 0.030 | 0.013 | 0.019 |
| rs6499084 | A | G | 0.354 | 0.007 | 0.001 | 5.6e-11 | 43.0 | 0.003 | 0.013 | 0.792 |
| rs6502670 | T | G | 0.617 | -0.007 | 0.001 | 7.6e-12 | 46.8 | -0.001 | 0.012 | 0.918 |
| rs6534338 | T | C | 0.298 | 0.010 | 0.001 | 4.1e-20 | 84.3 | 0.035 | 0.013 | 0.005 |
| rs6534704 | A | T | 0.073 | -0.015 | 0.002 | 2.0e-15 | 63.0 | 0.009 | 0.024 | 0.715 |
| rs6539284 | T | C | 0.613 | -0.007 | 0.001 | 1.4e-11 | 45.4 | -0.008 | 0.013 | 0.533 |
| rs6545559 | T | C | 0.317 | -0.008 | 0.001 | 8.2e-12 | 46.9 | -0.008 | 0.014 | 0.589 |
| rs6547396 | T | C | 0.580 | 0.008 | 0.001 | 4.5e-13 | 52.1 | 0.013 | 0.013 | 0.291 |
| rs6563363 | T | G | 0.357 | -0.009 | 0.001 | 1.7e-17 | 72.7 | 0.012 | 0.013 | 0.337 |
| rs6580068 | T | C | 0.225 | -0.009 | 0.001 | 2.0e-13 | 53.7 | 0.003 | 0.014 | 0.844 |
| rs660010 | C | G | 0.124 | 0.019 | 0.002 | 3.2e-35 | 154.3 | 0.008 | 0.019 | 0.652 |
| rs66671632 | T | C | 0.153 | -0.010 | 0.002 | 1.4e-11 | 45.6 | 0.017 | 0.021 | 0.423 |
| rs669952 | A | G | 0.182 | 0.008 | 0.001 | 6.3e-09 | 33.9 | -0.016 | 0.018 | 0.385 |
| rs67033024 | A | G | 0.848 | 0.009 | 0.002 | 9.8e-10 | 37.3 | -0.004 | 0.017 | 0.822 |
| rs67040074 | T | C | 0.353 | -0.012 | 0.001 | 2.3e-28 | 122.2 | -0.002 | 0.013 | 0.886 |
| rs6704241 | A | G | 0.679 | 0.009 | 0.001 | 8.4e-15 | 59.9 | 0.002 | 0.014 | 0.875 |
| rs6704768 | A | G | 0.542 | -0.015 | 0.001 | 9.7e-48 | 209.2 | 0.015 | 0.012 | 0.228 |
| rs6713695 | A | G | 0.458 | -0.011 | 0.001 | 5.2e-24 | 101.9 | 0.007 | 0.013 | 0.552 |
| rs6715321 | T | C | 0.439 | -0.010 | 0.001 | 2.2e-20 | 85.3 | 0.021 | 0.013 | 0.099 |
| rs6721505 | A | G | 0.325 | 0.007 | 0.001 | 4.1e-11 | 43.2 | -0.011 | 0.013 | 0.380 |
| rs6731373 | A | G | 0.320 | -0.009 | 0.001 | 2.6e-15 | 62.8 | -0.016 | 0.015 | 0.269 |
| rs6747129 | A | G | 0.119 | -0.016 | 0.002 | 1.1e-19 | 82.1 | -0.038 | 0.018 | 0.038 |
| rs6779981 | T | C | 0.599 | 0.007 | 0.001 | 2.4e-10 | 39.9 | -0.002 | 0.013 | 0.897 |
| rs6782698 | A | G | 0.758 | 0.014 | 0.001 | 4.8e-30 | 130.6 | 0.000 | 0.014 | 0.971 |
| rs67829508 | A | G | 0.176 | -0.009 | 0.001 | 1.2e-11 | 45.8 | -0.013 | 0.017 | 0.448 |
| rs6801153 | T | C | 0.247 | -0.009 | 0.001 | 2.1e-12 | 49.2 | 0.003 | 0.015 | 0.860 |
| rs6839051 | A | C | 0.383 | -0.007 | 0.001 | 1.4e-10 | 40.8 | 0.019 | 0.013 | 0.125 |
| rs6898748 | T | C | 0.560 | -0.007 | 0.001 | 3.6e-10 | 39.6 | -0.006 | 0.012 | 0.623 |
| rs6907381 | A | G | 0.131 | -0.012 | 0.002 | 9.1e-14 | 55.5 | -0.023 | 0.018 | 0.196 |
| rs6911986 | T | C | 0.269 | -0.008 | 0.001 | 2.5e-12 | 49.2 | -0.016 | 0.014 | 0.246 |
| rs6929638 | A | G | 0.512 | 0.009 | 0.001 | 6.2e-19 | 78.8 | 0.012 | 0.013 | 0.321 |
| rs6930903 | A | G | 0.738 | 0.016 | 0.001 | 4.7e-33 | 142.4 | -0.019 | 0.014 | 0.160 |
| rs6956241 | T | G | 0.145 | 0.013 | 0.002 | 5.9e-19 | 78.9 | 0.023 | 0.014 | 0.103 |
| rs6960056 | A | G | 0.451 | 0.013 | 0.001 | 3.5e-34 | 150.0 | 0.003 | 0.013 | 0.812 |
| rs7009856 | C | G | 0.404 | 0.007 | 0.001 | 4.1e-10 | 39.3 | 0.006 | 0.013 | 0.639 |
| rs7026972 | T | C | 0.649 | -0.012 | 0.001 | 7.1e-26 | 111.3 | -0.009 | 0.013 | 0.467 |
| rs7027019 | T | C | 0.677 | -0.006 | 0.001 | 3.3e-08 | 30.4 | -0.007 | 0.013 | 0.579 |
| rs7027899 | A | G | 0.809 | 0.011 | 0.002 | 3.8e-09 | 34.7 | -0.002 | 0.016 | 0.891 |
| rs7032484 | A | G | 0.569 | 0.013 | 0.001 | 6.8e-35 | 150.6 | -0.011 | 0.013 | 0.380 |
| rs7039819 | A | G | 0.581 | 0.009 | 0.001 | 4.4e-18 | 75.2 | -0.009 | 0.013 | 0.470 |
| rs705240 | T | C | 0.181 | -0.013 | 0.001 | 4.6e-22 | 92.9 | 0.011 | 0.014 | 0.450 |
| rs708912 | T | C | 0.780 | 0.010 | 0.001 | 9.0e-16 | 64.6 | 0.008 | 0.014 | 0.560 |
| rs7097348 | T | C | 0.725 | -0.009 | 0.001 | 6.4e-15 | 60.9 | -0.006 | 0.014 | 0.672 |
| rs7109373 | A | T | 0.806 | 0.010 | 0.001 | 2.9e-15 | 62.2 | -0.013 | 0.018 | 0.459 |
| rs7131691 | A | G | 0.484 | -0.007 | 0.001 | 1.1e-12 | 50.7 | 0.026 | 0.013 | 0.036 |
| rs71366589 | T | C | 0.984 | 0.023 | 0.004 | 1.5e-09 | 36.5 | 0.123 | 0.078 | 0.115 |
| rs7150195 | T | G | 0.365 | 0.014 | 0.001 | 1.4e-36 | 159.7 | 0.000 | 0.013 | 0.979 |
| rs7151326 | T | G | 0.412 | -0.009 | 0.001 | 5.7e-16 | 66.1 | 0.011 | 0.013 | 0.372 |
| rs7172133 | A | C | 0.689 | 0.007 | 0.001 | 4.9e-11 | 43.1 | -0.013 | 0.013 | 0.298 |
| rs717997 | A | G | 0.421 | 0.013 | 0.001 | 2.5e-34 | 149.7 | 0.001 | 0.013 | 0.958 |
| rs7184863 | T | G | 0.802 | 0.008 | 0.001 | 3.7e-09 | 34.9 | 0.007 | 0.015 | 0.650 |
| rs7236339 | A | G | 0.203 | -0.015 | 0.001 | 1.6e-33 | 145.3 | -0.010 | 0.014 | 0.482 |
| rs7240432 | T | G | 0.361 | 0.010 | 0.001 | 1.7e-21 | 90.9 | -0.008 | 0.013 | 0.550 |
| rs7254263 | T | C | 0.295 | -0.010 | 0.001 | 7.9e-18 | 74.6 | -0.024 | 0.015 | 0.101 |
| rs72672601 | A | G | 0.579 | -0.010 | 0.001 | 2.0e-19 | 80.8 | -0.014 | 0.012 | 0.243 |
| rs72674843 | T | C | 0.725 | -0.011 | 0.001 | 1.0e-18 | 78.1 | -0.006 | 0.013 | 0.647 |
| rs72695265 | T | C | 0.226 | -0.010 | 0.001 | 1.1e-14 | 60.2 | -0.010 | 0.014 | 0.486 |
| rs72717150 | A | G | 0.069 | -0.019 | 0.002 | 1.7e-22 | 95.2 | -0.018 | 0.027 | 0.494 |
| rs72725852 | T | C | 0.316 | 0.007 | 0.001 | 3.3e-09 | 35.0 | 0.011 | 0.013 | 0.416 |
| rs72748123 | A | G | 0.160 | -0.012 | 0.001 | 7.7e-17 | 69.1 | 0.011 | 0.017 | 0.498 |
| rs72778512 | C | G | 0.913 | -0.012 | 0.002 | 3.8e-11 | 43.7 | 0.002 | 0.021 | 0.932 |
| rs72801843 | A | T | 0.292 | 0.015 | 0.001 | 1.8e-40 | 177.5 | 0.004 | 0.014 | 0.780 |
| rs72819955 | T | C | 0.025 | -0.025 | 0.003 | 4.9e-16 | 65.8 | -0.017 | 0.041 | 0.685 |
| rs72829857 | A | G | 0.760 | -0.017 | 0.001 | 7.3e-45 | 198.6 | -0.003 | 0.017 | 0.885 |
| rs72838750 | T | G | 0.502 | -0.009 | 0.001 | 8.3e-17 | 69.8 | -0.008 | 0.012 | 0.503 |
| rs72890842 | T | G | 0.765 | 0.013 | 0.001 | 4.8e-25 | 106.7 | -0.006 | 0.015 | 0.675 |
| rs72906124 | T | C | 0.948 | 0.018 | 0.002 | 4.9e-16 | 66.0 | -0.010 | 0.036 | 0.788 |
| rs72915557 | A | G | 0.052 | -0.021 | 0.002 | 1.2e-20 | 86.6 | 0.007 | 0.044 | 0.880 |
| rs7296742 | A | G | 0.274 | -0.009 | 0.001 | 2.1e-13 | 54.3 | 0.003 | 0.017 | 0.849 |
| rs72993796 | T | C | 0.892 | 0.015 | 0.002 | 8.6e-20 | 83.0 | 0.003 | 0.020 | 0.894 |
| rs7301232 | A | T | 0.168 | -0.008 | 0.001 | 3.8e-08 | 30.5 | 0.019 | 0.018 | 0.301 |
| rs7302808 | T | C | 0.481 | 0.006 | 0.001 | 1.2e-09 | 36.6 | 0.021 | 0.012 | 0.097 |
| rs7304399 | A | G | 0.458 | 0.011 | 0.001 | 2.2e-25 | 108.4 | -0.026 | 0.013 | 0.035 |
| rs73055556 | A | G | 0.136 | 0.011 | 0.002 | 2.5e-13 | 53.5 | -0.015 | 0.020 | 0.465 |
| rs7306755 | A | G | 0.198 | 0.019 | 0.001 | 2.5e-48 | 212.7 | 0.015 | 0.015 | 0.311 |
| rs73117392 | T | C | 0.948 | -0.023 | 0.002 | 3.6e-24 | 102.8 | -0.002 | 0.033 | 0.964 |
| rs7312511 | T | C | 0.105 | -0.010 | 0.002 | 2.5e-08 | 30.9 | 0.026 | 0.022 | 0.241 |
| rs73141547 | A | T | 0.659 | -0.016 | 0.001 | 1.3e-45 | 201.4 | -0.008 | 0.013 | 0.508 |
| rs73210523 | A | G | 0.165 | 0.012 | 0.001 | 9.7e-18 | 73.6 | -0.025 | 0.017 | 0.130 |
| rs73219806 | A | C | 0.172 | 0.012 | 0.001 | 1.3e-18 | 77.3 | 0.012 | 0.017 | 0.490 |
| rs7328289 | T | C | 0.794 | -0.007 | 0.001 | 3.3e-08 | 30.6 | -0.016 | 0.017 | 0.345 |
| rs7338804 | A | G | 0.143 | -0.008 | 0.002 | 2.3e-08 | 31.4 | -0.002 | 0.019 | 0.935 |
| rs7356921 | T | C | 0.246 | -0.015 | 0.001 | 9.1e-34 | 147.6 | -0.016 | 0.014 | 0.234 |
| rs738988 | A | G | 0.738 | -0.016 | 0.001 | 1.4e-43 | 191.4 | 0.003 | 0.016 | 0.835 |
| rs7430651 | T | C | 0.274 | -0.011 | 0.001 | 2.2e-22 | 94.2 | -0.017 | 0.014 | 0.229 |
| rs7449561 | A | G | 0.234 | 0.010 | 0.001 | 6.8e-15 | 60.6 | -0.001 | 0.015 | 0.959 |
| rs74677779 | C | G | 0.956 | -0.016 | 0.003 | 1.2e-09 | 36.9 | 0.004 | 0.024 | 0.875 |
| rs7469569 | T | C | 0.346 | 0.010 | 0.001 | 3.6e-18 | 75.1 | 0.018 | 0.013 | 0.163 |
| rs74740938 | A | T | 0.132 | 0.012 | 0.002 | 8.3e-14 | 55.6 | 0.011 | 0.022 | 0.608 |
| rs748832 | A | G | 0.638 | 0.010 | 0.001 | 1.6e-20 | 86.9 | -0.009 | 0.013 | 0.488 |
| rs7515450 | A | T | 0.411 | 0.008 | 0.001 | 7.3e-14 | 56.2 | -0.013 | 0.013 | 0.308 |
| rs7520927 | A | G | 0.478 | -0.007 | 0.001 | 6.4e-11 | 42.4 | 0.000 | 0.012 | 0.991 |
| rs75624576 | T | G | 0.096 | 0.015 | 0.002 | 1.4e-18 | 77.0 | 0.030 | 0.019 | 0.113 |
| rs7578247 | A | G | 0.609 | -0.008 | 0.001 | 2.3e-13 | 53.6 | 0.013 | 0.013 | 0.352 |
| rs75795256 | T | C | 0.910 | -0.014 | 0.002 | 1.0e-13 | 55.2 | 0.035 | 0.026 | 0.182 |
| rs7603132 | A | G | 0.184 | 0.017 | 0.001 | 2.7e-38 | 166.5 | 0.000 | 0.015 | 0.988 |
| rs76076331 | T | C | 0.123 | 0.022 | 0.002 | 1.5e-45 | 200.0 | 0.037 | 0.016 | 0.020 |
| rs7626224 | T | G | 0.762 | 0.008 | 0.001 | 1.4e-10 | 41.3 | 0.010 | 0.015 | 0.500 |
| rs7632819 | A | G | 0.761 | -0.013 | 0.001 | 1.1e-26 | 113.7 | -0.043 | 0.014 | 0.002 |
| rs7650602 | T | C | 0.568 | -0.010 | 0.001 | 3.2e-21 | 88.6 | 0.010 | 0.012 | 0.445 |
| rs7668960 | T | C | 0.170 | 0.016 | 0.001 | 5.4e-28 | 119.8 | -0.041 | 0.019 | 0.029 |
| rs76878669 | C | G | 0.767 | 0.012 | 0.001 | 6.5e-23 | 97.1 | -0.002 | 0.014 | 0.871 |
| rs77128898 | T | C | 0.031 | -0.024 | 0.003 | 2.1e-16 | 67.3 | 0.043 | 0.028 | 0.131 |
| rs77187837 | T | C | 0.123 | 0.013 | 0.002 | 5.8e-16 | 65.3 | -0.001 | 0.019 | 0.954 |
| rs77584294 | A | C | 0.037 | 0.026 | 0.003 | 3.9e-22 | 93.5 | 0.035 | 0.046 | 0.442 |
| rs7762296 | T | C | 0.312 | -0.008 | 0.001 | 2.0e-13 | 53.6 | -0.007 | 0.014 | 0.604 |
| rs77882218 | T | C | 0.970 | -0.026 | 0.003 | 4.1e-16 | 66.2 | -0.018 | 0.034 | 0.602 |
| rs7788620 | A | G | 0.786 | 0.017 | 0.001 | 8.3e-40 | 175.7 | -0.025 | 0.017 | 0.143 |
| rs77899607 | T | C | 0.108 | 0.013 | 0.002 | 6.5e-17 | 69.4 | 0.020 | 0.019 | 0.283 |
| rs77926719 | A | G | 0.107 | 0.011 | 0.002 | 7.2e-12 | 47.0 | 0.010 | 0.019 | 0.606 |
| rs78116078 | C | G | 0.726 | 0.009 | 0.001 | 1.3e-15 | 64.3 | -0.003 | 0.014 | 0.814 |
| rs78280128 | A | G | 0.920 | -0.020 | 0.002 | 2.0e-26 | 113.1 | 0.024 | 0.020 | 0.225 |
| rs78410329 | T | C | 0.237 | 0.018 | 0.001 | 1.6e-45 | 200.2 | 0.002 | 0.013 | 0.887 |
| rs78462816 | A | G | 0.888 | -0.010 | 0.002 | 1.4e-10 | 41.0 | 0.009 | 0.021 | 0.677 |
| rs786244 | C | G | 0.341 | 0.010 | 0.001 | 6.1e-21 | 88.4 | 0.029 | 0.013 | 0.032 |
| rs792213 | A | C | 0.412 | 0.007 | 0.001 | 5.3e-12 | 47.8 | 0.006 | 0.013 | 0.612 |
| rs7924036 | T | G | 0.505 | 0.014 | 0.001 | 1.9e-41 | 180.8 | 0.010 | 0.012 | 0.440 |
| rs7924465 | T | C | 0.909 | 0.014 | 0.002 | 8.5e-16 | 64.7 | 0.001 | 0.024 | 0.973 |
| rs7933521 | T | C | 0.839 | 0.008 | 0.001 | 2.9e-08 | 30.7 | 0.015 | 0.016 | 0.345 |
| rs7936656 | A | G | 0.314 | -0.008 | 0.001 | 1.1e-13 | 55.3 | 0.012 | 0.013 | 0.374 |
| rs795540 | T | C | 0.591 | -0.008 | 0.001 | 6.0e-14 | 56.7 | 0.007 | 0.013 | 0.567 |
| rs7977614 | A | G | 0.725 | -0.011 | 0.001 | 2.5e-21 | 89.3 | -0.007 | 0.014 | 0.638 |
| rs7979979 | T | C | 0.227 | -0.012 | 0.001 | 1.2e-21 | 91.2 | -0.022 | 0.017 | 0.205 |
| rs7983020 | T | C | 0.703 | -0.009 | 0.001 | 1.3e-14 | 59.3 | 0.011 | 0.013 | 0.391 |
| rs79925479 | T | G | 0.052 | 0.014 | 0.002 | 3.6e-09 | 35.0 | -0.004 | 0.036 | 0.901 |
| rs80049472 | A | G | 0.104 | -0.010 | 0.002 | 1.2e-08 | 32.5 | 0.008 | 0.019 | 0.661 |
| rs80171383 | A | G | 0.125 | 0.015 | 0.002 | 1.2e-21 | 91.3 | 0.005 | 0.018 | 0.766 |
| rs8020023 | A | G | 0.247 | 0.014 | 0.001 | 6.3e-32 | 138.0 | 0.003 | 0.016 | 0.832 |
| rs80257979 | T | G | 0.970 | -0.026 | 0.003 | 5.8e-19 | 79.1 | -0.009 | 0.031 | 0.761 |
| rs80352808 | A | G | 0.287 | -0.008 | 0.001 | 1.1e-10 | 41.9 | 0.007 | 0.014 | 0.621 |
| rs8044562 | A | G | 0.277 | -0.012 | 0.001 | 8.3e-25 | 105.8 | 0.012 | 0.013 | 0.372 |
| rs8058137 | A | G | 0.773 | 0.015 | 0.001 | 8.6e-31 | 133.2 | 0.013 | 0.014 | 0.366 |
| rs8072494 | A | G | 0.789 | -0.012 | 0.001 | 1.6e-19 | 82.1 | 0.017 | 0.017 | 0.325 |
| rs8110747 | T | C | 0.389 | -0.007 | 0.001 | 6.0e-10 | 38.7 | -0.005 | 0.013 | 0.681 |
| rs837080 | T | C | 0.535 | -0.010 | 0.001 | 1.1e-20 | 86.6 | -0.014 | 0.013 | 0.269 |
| rs853199 | T | C | 0.332 | 0.009 | 0.001 | 3.8e-15 | 62.1 | 0.007 | 0.013 | 0.588 |
| rs863006 | A | G | 0.558 | -0.010 | 0.001 | 1.3e-20 | 86.2 | -0.004 | 0.012 | 0.737 |
| rs872602 | A | G | 0.249 | -0.013 | 0.001 | 1.1e-24 | 104.8 | 0.005 | 0.014 | 0.732 |
| rs899223 | C | G | 0.079 | 0.013 | 0.002 | 5.1e-12 | 47.6 | 0.015 | 0.020 | 0.451 |
| rs903981 | T | G | 0.431 | -0.007 | 0.001 | 1.7e-11 | 45.5 | -0.005 | 0.013 | 0.676 |
| rs912609 | T | G | 0.240 | 0.012 | 0.001 | 6.6e-23 | 97.6 | 0.006 | 0.013 | 0.672 |
| rs9317202 | T | C | 0.699 | 0.010 | 0.001 | 1.3e-17 | 73.7 | -0.002 | 0.013 | 0.855 |
| rs9356034 | T | C | 0.669 | 0.010 | 0.001 | 4.7e-20 | 83.7 | 0.001 | 0.013 | 0.942 |
| rs9359939 | A | C | 0.238 | -0.011 | 0.001 | 6.6e-19 | 79.1 | -0.001 | 0.013 | 0.927 |
| rs9372734 | T | C | 0.464 | 0.022 | 0.001 | 3.1e-97 | 435.4 | 0.027 | 0.012 | 0.031 |
| rs9374194 | T | C | 0.809 | -0.009 | 0.001 | 4.1e-11 | 43.9 | 0.016 | 0.015 | 0.277 |
| rs9388490 | T | C | 0.472 | 0.012 | 0.001 | 8.4e-28 | 120.0 | 0.015 | 0.012 | 0.244 |
| rs9411336 | T | C | 0.337 | -0.013 | 0.001 | 1.6e-30 | 131.6 | -0.016 | 0.014 | 0.254 |
| rs9461242 | T | C | 0.351 | -0.014 | 0.001 | 4.6e-36 | 156.1 | -0.014 | 0.013 | 0.311 |
| rs9496630 | A | T | 0.277 | -0.007 | 0.001 | 1.3e-08 | 32.6 | 0.015 | 0.014 | 0.291 |
| rs952062 | A | T | 0.127 | 0.010 | 0.002 | 1.3e-09 | 36.6 | 0.003 | 0.017 | 0.846 |
| rs9537509 | T | C | 0.744 | -0.007 | 0.001 | 5.3e-09 | 34.1 | -0.011 | 0.015 | 0.444 |
| rs9540731 | T | C | 0.501 | 0.011 | 0.001 | 9.9e-25 | 104.8 | -0.001 | 0.012 | 0.966 |
| rs9540920 | A | G | 0.579 | 0.006 | 0.001 | 3.4e-08 | 30.4 | -0.005 | 0.013 | 0.733 |
| rs9551887 | T | C | 0.531 | -0.008 | 0.001 | 2.9e-14 | 58.0 | -0.012 | 0.012 | 0.324 |
| rs9555651 | T | C | 0.155 | 0.011 | 0.001 | 3.0e-15 | 62.4 | 0.009 | 0.018 | 0.605 |
| rs9635366 | A | G | 0.211 | 0.016 | 0.001 | 2.0e-32 | 140.1 | -0.023 | 0.014 | 0.110 |
| rs9652254 | A | G | 0.603 | 0.006 | 0.001 | 1.0e-09 | 37.5 | 0.025 | 0.013 | 0.054 |
| rs975303 | A | G | 0.839 | -0.015 | 0.001 | 1.2e-26 | 114.6 | 0.033 | 0.017 | 0.058 |
| rs9787076 | A | C | 0.681 | -0.018 | 0.001 | 3.5e-61 | 274.2 | 0.000 | 0.013 | 0.990 |
| rs9859719 | A | G | 0.211 | -0.011 | 0.001 | 3.7e-19 | 80.1 | 0.005 | 0.016 | 0.744 |
| rs9871964 | T | C | 0.181 | -0.010 | 0.001 | 5.4e-12 | 47.5 | 0.033 | 0.017 | 0.048 |
| rs9899056 | A | G | 0.397 | 0.007 | 0.001 | 6.7e-11 | 42.6 | -0.008 | 0.013 | 0.551 |
| rs989996 | T | C | 0.439 | -0.009 | 0.001 | 3.0e-19 | 80.5 | 0.001 | 0.012 | 0.940 |
| rs9914251 | A | G | 0.751 | 0.008 | 0.001 | 3.0e-10 | 39.8 | 0.008 | 0.014 | 0.576 |
| rs9922788 | A | G | 0.408 | 0.010 | 0.001 | 2.1e-22 | 94.1 | -0.007 | 0.012 | 0.601 |
| rs9930253 | A | T | 0.281 | -0.010 | 0.001 | 3.1e-17 | 71.0 | -0.007 | 0.014 | 0.652 |
| rs9937449 | T | C | 0.551 | 0.008 | 0.001 | 1.6e-13 | 54.8 | -0.015 | 0.012 | 0.220 |
| rs9985296 | T | C | 0.384 | -0.010 | 0.001 | 9.9e-22 | 91.1 | 0.004 | 0.012 | 0.735 |
| rs9993133 | T | C | 0.810 | 0.008 | 0.001 | 3.5e-08 | 30.4 | 0.016 | 0.016 | 0.330 |
| EA, effect allele. OA, other allele. EAF, effect allele frequency. SE, standard error. | | | | | | | | | | |

**Supplementary Table 3** Heterogeneity of Wald ratios and MR-Egger test for directional pleiotropy

|  | Heterogeneity | | |  |
| --- | --- | --- | --- | --- |
|  | Q | Degrees of Freedom | P | I_GX_^2^ |
| Educational attainment | 616.22 | 560 | 0.05 | 0.091 |
|  | MR-Egger test for directional pleiotropy | | |  |
|  | Intercept | Standard error | P |  |
| Educational attainment | 1.908e-03 | 0.002 | 0.328 |  |

**Supplementary Table 4** Inverse variance weighted estimates in leave-one-out analysis

| SNP excluded | SNP | OR | (95% CI) | P value |
| --- | --- | --- | --- | --- |
| Educational attainment | rs10014934 | 1.52 | (1.03;2.23) | 0.033 |
|  | rs10016110 | 1.49 | (1.02;2.19) | 0.041 |
|  | rs10063055 | 1.52 | (1.03;2.23) | 0.034 |
|  | rs10167909 | 1.51 | (1.03;2.21) | 0.036 |
|  | rs10180485 | 1.50 | (1.02;2.21) | 0.038 |
|  | rs10180845 | 1.51 | (1.03;2.22) | 0.036 |
|  | rs10186870 | 1.51 | (1.03;2.21) | 0.036 |
|  | rs10189857 | 1.48 | (1.01;2.17) | 0.047 |
|  | rs10193498 | 1.51 | (1.03;2.22) | 0.035 |
|  | rs10202640 | 1.51 | (1.03;2.21) | 0.037 |
|  | rs10240905 | 1.49 | (1.02;2.19) | 0.041 |
|  | rs10256396 | 1.49 | (1.02;2.19) | 0.041 |
|  | rs10283803 | 1.51 | (1.03;2.22) | 0.035 |
|  | rs10401329 | 1.51 | (1.03;2.21) | 0.036 |
|  | rs1043595 | 1.49 | (1.01;2.19) | 0.042 |
|  | rs10481106 | 1.50 | (1.02;2.2) | 0.038 |
|  | rs10496632 | 1.52 | (1.03;2.23) | 0.034 |
|  | rs10507085 | 1.50 | (1.02;2.2) | 0.039 |
|  | rs1056010 | 1.50 | (1.02;2.2) | 0.039 |
|  | rs1058790 | 1.50 | (1.02;2.21) | 0.038 |
|  | rs10732635 | 1.49 | (1.02;2.19) | 0.040 |
|  | rs10734924 | 1.50 | (1.02;2.2) | 0.040 |
|  | rs10739569 | 1.50 | (1.02;2.21) | 0.037 |
|  | rs10740572 | 1.49 | (1.02;2.19) | 0.040 |
|  | rs10745307 | 1.53 | (1.04;2.24) | 0.029 |
|  | rs10748559 | 1.50 | (1.02;2.21) | 0.037 |
|  | rs10752262 | 1.51 | (1.03;2.22) | 0.035 |
|  | rs10763979 | 1.48 | (1.01;2.17) | 0.045 |
|  | rs10765789 | 1.50 | (1.02;2.21) | 0.037 |
|  | rs10772644 | 1.49 | (1.02;2.19) | 0.041 |
|  | rs10815961 | 1.50 | (1.02;2.21) | 0.037 |
|  | rs10819974 | 1.50 | (1.02;2.2) | 0.038 |
|  | rs10835389 | 1.50 | (1.02;2.2) | 0.040 |
|  | rs10853981 | 1.50 | (1.02;2.2) | 0.040 |
|  | rs10865834 | 1.50 | (1.02;2.2) | 0.038 |
|  | rs10867548 | 1.51 | (1.03;2.22) | 0.035 |
|  | rs10874759 | 1.50 | (1.02;2.2) | 0.040 |
|  | rs10887465 | 1.52 | (1.04;2.24) | 0.032 |
|  | rs10887592 | 1.51 | (1.03;2.21) | 0.036 |
|  | rs10895719 | 1.50 | (1.02;2.2) | 0.039 |
|  | rs10929474 | 1.50 | (1.02;2.21) | 0.038 |
|  | rs11019128 | 1.51 | (1.03;2.22) | 0.034 |
|  | rs11028323 | 1.49 | (1.02;2.19) | 0.042 |
|  | rs11081529 | 1.50 | (1.02;2.21) | 0.038 |
|  | rs11138947 | 1.51 | (1.03;2.22) | 0.035 |
|  | rs11157931 | 1.52 | (1.03;2.23) | 0.034 |
|  | rs11158200 | 1.50 | (1.02;2.2) | 0.039 |
|  | rs11159067 | 1.47 | (1;2.15) | 0.048 |
|  | rs11172371 | 1.49 | (1.01;2.18) | 0.043 |
|  | rs11181124 | 1.51 | (1.03;2.22) | 0.035 |
|  | rs111821073 | 1.48 | (1.01;2.18) | 0.044 |
|  | rs11191193 | 1.50 | (1.02;2.2) | 0.040 |
|  | rs11196397 | 1.50 | (1.02;2.2) | 0.039 |
|  | rs11218422 | 1.51 | (1.03;2.22) | 0.035 |
|  | rs112255786 | 1.52 | (1.04;2.24) | 0.031 |
|  | rs11245450 | 1.49 | (1.02;2.19) | 0.041 |
|  | rs1125854 | 1.50 | (1.02;2.21) | 0.038 |
|  | rs11264531 | 1.49 | (1.02;2.19) | 0.041 |
|  | rs112780312 | 1.48 | (1.01;2.18) | 0.044 |
|  | rs1128687 | 1.51 | (1.03;2.21) | 0.037 |
|  | rs1128956 | 1.51 | (1.03;2.22) | 0.036 |
|  | rs113389222 | 1.51 | (1.03;2.21) | 0.036 |
|  | rs114440021 | 1.49 | (1.02;2.19) | 0.040 |
|  | rs114468556 | 1.51 | (1.03;2.22) | 0.034 |
|  | rs115003386 | 1.51 | (1.03;2.21) | 0.036 |
|  | rs115124024 | 1.51 | (1.03;2.21) | 0.035 |
|  | rs1156541 | 1.49 | (1.02;2.19) | 0.041 |
|  | rs11571404 | 1.52 | (1.04;2.23) | 0.032 |
|  | rs116068530 | 1.51 | (1.03;2.22) | 0.035 |
|  | rs11614957 | 1.52 | (1.04;2.24) | 0.032 |
|  | rs116173579 | 1.51 | (1.03;2.22) | 0.034 |
|  | rs11633934 | 1.50 | (1.02;2.2) | 0.040 |
|  | rs11643516 | 1.50 | (1.02;2.21) | 0.037 |
|  | rs11664320 | 1.53 | (1.04;2.24) | 0.031 |
|  | rs1167796 | 1.51 | (1.03;2.22) | 0.034 |
|  | rs117005905 | 1.51 | (1.03;2.22) | 0.034 |
|  | rs11724690 | 1.51 | (1.03;2.22) | 0.036 |
|  | rs11746390 | 1.50 | (1.02;2.2) | 0.039 |
|  | rs117494884 | 1.51 | (1.03;2.22) | 0.034 |
|  | rs117520198 | 1.50 | (1.02;2.21) | 0.038 |
|  | rs117520996 | 1.51 | (1.03;2.22) | 0.036 |
|  | rs11757278 | 1.50 | (1.02;2.21) | 0.038 |
|  | rs11772108 | 1.51 | (1.03;2.22) | 0.035 |
|  | rs11773992 | 1.49 | (1.02;2.19) | 0.041 |
|  | rs11774212 | 1.52 | (1.03;2.23) | 0.034 |
|  | rs118134876 | 1.49 | (1.02;2.19) | 0.042 |
|  | rs1182532 | 1.49 | (1.01;2.19) | 0.042 |
|  | rs11861256 | 1.50 | (1.02;2.2) | 0.039 |
|  | rs11925699 | 1.49 | (1.02;2.19) | 0.040 |
|  | rs1193240 | 1.50 | (1.02;2.21) | 0.037 |
|  | rs11942352 | 1.50 | (1.02;2.2) | 0.039 |
|  | rs12055782 | 1.52 | (1.03;2.23) | 0.034 |
|  | rs12089815 | 1.50 | (1.02;2.2) | 0.040 |
|  | rs12113634 | 1.48 | (1.01;2.16) | 0.046 |
|  | rs12126231 | 1.50 | (1.02;2.21) | 0.038 |
|  | rs12142570 | 1.50 | (1.02;2.21) | 0.037 |
|  | rs12150824 | 1.50 | (1.02;2.2) | 0.039 |
|  | rs12151015 | 1.52 | (1.03;2.22) | 0.034 |
|  | rs12155540 | 1.51 | (1.03;2.21) | 0.037 |
|  | rs12201073 | 1.49 | (1.02;2.19) | 0.040 |
|  | rs12234936 | 1.53 | (1.04;2.24) | 0.031 |
|  | rs12240387 | 1.50 | (1.02;2.21) | 0.037 |
|  | rs12273435 | 1.51 | (1.03;2.21) | 0.036 |
|  | rs12339610 | 1.50 | (1.02;2.21) | 0.037 |
|  | rs12362273 | 1.49 | (1.02;2.19) | 0.041 |
|  | rs12431579 | 1.50 | (1.02;2.2) | 0.038 |
|  | rs12435895 | 1.49 | (1.02;2.19) | 0.041 |
|  | rs12506222 | 1.48 | (1.01;2.18) | 0.044 |
|  | rs12524795 | 1.49 | (1.02;2.19) | 0.041 |
|  | rs12569163 | 1.49 | (1.02;2.19) | 0.041 |
|  | rs12609965 | 1.50 | (1.02;2.2) | 0.039 |
|  | rs12632133 | 1.50 | (1.02;2.21) | 0.038 |
|  | rs12699131 | 1.53 | (1.04;2.24) | 0.030 |
|  | rs12709690 | 1.49 | (1.02;2.19) | 0.042 |
|  | rs12764593 | 1.52 | (1.04;2.23) | 0.033 |
|  | rs12768641 | 1.52 | (1.03;2.22) | 0.034 |
|  | rs1278537 | 1.51 | (1.03;2.22) | 0.035 |
|  | rs12828220 | 1.49 | (1.01;2.18) | 0.043 |
|  | rs12893970 | 1.50 | (1.02;2.2) | 0.039 |
|  | rs12897763 | 1.48 | (1.01;2.17) | 0.047 |
|  | rs12915175 | 1.51 | (1.03;2.22) | 0.034 |
|  | rs1291865 | 1.49 | (1.01;2.18) | 0.043 |
|  | rs12937411 | 1.53 | (1.04;2.24) | 0.031 |
|  | rs12962980 | 1.52 | (1.03;2.23) | 0.034 |
|  | rs12974657 | 1.49 | (1.01;2.18) | 0.043 |
|  | rs12981405 | 1.50 | (1.02;2.2) | 0.038 |
|  | rs13010288 | 1.50 | (1.02;2.2) | 0.039 |
|  | rs13030436 | 1.53 | (1.04;2.24) | 0.030 |
|  | rs13122283 | 1.51 | (1.03;2.21) | 0.036 |
|  | rs13133213 | 1.49 | (1.02;2.19) | 0.041 |
|  | rs13135092 | 1.52 | (1.04;2.23) | 0.031 |
|  | rs13138842 | 1.49 | (1.02;2.19) | 0.041 |
|  | rs13159124 | 1.50 | (1.02;2.21) | 0.038 |
|  | rs13161488 | 1.52 | (1.03;2.22) | 0.034 |
|  | rs13212041 | 1.48 | (1.01;2.18) | 0.044 |
|  | rs13214394 | 1.49 | (1.02;2.19) | 0.040 |
|  | rs13244634 | 1.51 | (1.03;2.21) | 0.037 |
|  | rs13252156 | 1.52 | (1.03;2.23) | 0.033 |
|  | rs13278836 | 1.51 | (1.03;2.22) | 0.036 |
|  | rs13284474 | 1.50 | (1.02;2.2) | 0.038 |
|  | rs1329125 | 1.53 | (1.04;2.24) | 0.030 |
|  | rs13332638 | 1.50 | (1.02;2.2) | 0.039 |
|  | rs1334297 | 1.52 | (1.03;2.23) | 0.035 |
|  | rs1337731 | 1.51 | (1.03;2.22) | 0.034 |
|  | rs137121 | 1.51 | (1.03;2.21) | 0.036 |
|  | rs1378893 | 1.50 | (1.02;2.21) | 0.037 |
|  | rs1387148 | 1.50 | (1.02;2.21) | 0.037 |
|  | rs1392816 | 1.51 | (1.03;2.22) | 0.035 |
|  | rs139872934 | 1.50 | (1.02;2.2) | 0.040 |
|  | rs1407350 | 1.51 | (1.03;2.21) | 0.036 |
|  | rs141503359 | 1.52 | (1.03;2.23) | 0.033 |
|  | rs14184 | 1.51 | (1.02;2.21) | 0.037 |
|  | rs1452366 | 1.50 | (1.02;2.2) | 0.040 |
|  | rs145481578 | 1.51 | (1.03;2.21) | 0.036 |
|  | rs1464018 | 1.51 | (1.03;2.21) | 0.037 |
|  | rs1483148 | 1.49 | (1.02;2.19) | 0.041 |
|  | rs1483814 | 1.49 | (1.02;2.19) | 0.041 |
|  | rs1485272 | 1.50 | (1.02;2.2) | 0.039 |
|  | rs1490834 | 1.50 | (1.02;2.21) | 0.038 |
|  | rs149285517 | 1.49 | (1.02;2.19) | 0.041 |
|  | rs150121450 | 1.50 | (1.02;2.21) | 0.037 |
|  | rs1516036 | 1.51 | (1.03;2.21) | 0.036 |
|  | rs1585524 | 1.50 | (1.02;2.21) | 0.037 |
|  | rs158839 | 1.50 | (1.02;2.2) | 0.040 |
|  | rs1632822 | 1.50 | (1.02;2.2) | 0.039 |
|  | rs165633 | 1.50 | (1.02;2.2) | 0.039 |
|  | rs16844417 | 1.50 | (1.02;2.2) | 0.038 |
|  | rs1689510 | 1.47 | (1;2.15) | 0.051 |
|  | rs16919774 | 1.51 | (1.03;2.21) | 0.036 |
|  | rs16962221 | 1.50 | (1.02;2.2) | 0.039 |
|  | rs16983844 | 1.52 | (1.04;2.23) | 0.032 |
|  | rs17088142 | 1.49 | (1.02;2.19) | 0.041 |
|  | rs17096452 | 1.50 | (1.02;2.2) | 0.039 |
|  | rs17126938 | 1.50 | (1.02;2.21) | 0.038 |
|  | rs171697 | 1.52 | (1.04;2.24) | 0.032 |
|  | rs17224289 | 1.53 | (1.04;2.24) | 0.029 |
|  | rs17248751 | 1.47 | (1;2.15) | 0.048 |
|  | rs17257579 | 1.48 | (1.01;2.17) | 0.044 |
|  | rs17266097 | 1.52 | (1.04;2.23) | 0.032 |
|  | rs1738050 | 1.50 | (1.02;2.2) | 0.038 |
|  | rs17503473 | 1.49 | (1.02;2.19) | 0.041 |
|  | rs17563464 | 1.51 | (1.03;2.22) | 0.035 |
|  | rs17565975 | 1.50 | (1.02;2.2) | 0.039 |
|  | rs17649841 | 1.49 | (1.02;2.19) | 0.041 |
|  | rs17669337 | 1.49 | (1.02;2.19) | 0.041 |
|  | rs17721326 | 1.49 | (1.01;2.18) | 0.042 |
|  | rs17812330 | 1.49 | (1.02;2.19) | 0.040 |
|  | rs1786313 | 1.49 | (1.02;2.19) | 0.041 |
|  | rs17881016 | 1.52 | (1.03;2.23) | 0.033 |
|  | rs181164682 | 1.50 | (1.02;2.2) | 0.038 |
|  | rs1820986 | 1.49 | (1.02;2.19) | 0.041 |
|  | rs1835388 | 1.50 | (1.02;2.2) | 0.040 |
|  | rs184654 | 1.48 | (1.01;2.17) | 0.046 |
|  | rs1861786 | 1.51 | (1.03;2.22) | 0.035 |
|  | rs1865880 | 1.51 | (1.03;2.22) | 0.035 |
|  | rs1866710 | 1.55 | (1.06;2.27) | 0.025 |
|  | rs1866823 | 1.52 | (1.03;2.23) | 0.033 |
|  | rs1871745 | 1.52 | (1.03;2.22) | 0.034 |
|  | rs1880692 | 1.49 | (1.02;2.19) | 0.041 |
|  | rs1884368 | 1.52 | (1.03;2.23) | 0.033 |
|  | rs1888765 | 1.52 | (1.04;2.23) | 0.032 |
|  | rs192230289 | 1.51 | (1.03;2.21) | 0.036 |
|  | rs192436652 | 1.52 | (1.04;2.24) | 0.032 |
|  | rs1971218 | 1.49 | (1.01;2.18) | 0.042 |
|  | rs1980099 | 1.50 | (1.02;2.21) | 0.038 |
|  | rs2006853 | 1.50 | (1.02;2.2) | 0.040 |
|  | rs2026037 | 1.52 | (1.04;2.23) | 0.032 |
|  | rs2055940 | 1.50 | (1.02;2.2) | 0.038 |
|  | rs2063569 | 1.50 | (1.02;2.2) | 0.040 |
|  | rs211283 | 1.51 | (1.03;2.21) | 0.036 |
|  | rs213014 | 1.50 | (1.02;2.2) | 0.039 |
|  | rs2154209 | 1.51 | (1.03;2.22) | 0.035 |
|  | rs2160514 | 1.49 | (1.02;2.19) | 0.041 |
|  | rs2174752 | 1.52 | (1.03;2.23) | 0.033 |
|  | rs2177083 | 1.50 | (1.02;2.2) | 0.039 |
|  | rs2195041 | 1.51 | (1.03;2.21) | 0.036 |
|  | rs2210174 | 1.50 | (1.02;2.2) | 0.039 |
|  | rs2214631 | 1.49 | (1.02;2.19) | 0.040 |
|  | rs2216053 | 1.51 | (1.03;2.22) | 0.034 |
|  | rs2222760 | 1.50 | (1.02;2.21) | 0.037 |
|  | rs2238483 | 1.51 | (1.03;2.21) | 0.037 |
|  | rs2239736 | 1.51 | (1.03;2.21) | 0.037 |
|  | rs2275154 | 1.48 | (1;2.17) | 0.047 |
|  | rs2289769 | 1.51 | (1.03;2.22) | 0.035 |
|  | rs2297293 | 1.52 | (1.03;2.22) | 0.034 |
|  | rs2301015 | 1.51 | (1.03;2.21) | 0.036 |
|  | rs2303907 | 1.51 | (1.03;2.22) | 0.034 |
|  | rs2304282 | 1.53 | (1.05;2.25) | 0.029 |
|  | rs2352430 | 1.50 | (1.02;2.21) | 0.038 |
|  | rs2364972 | 1.49 | (1.01;2.18) | 0.042 |
|  | rs2371001 | 1.49 | (1.01;2.18) | 0.042 |
|  | rs2430813 | 1.51 | (1.03;2.22) | 0.034 |
|  | rs2447091 | 1.48 | (1.01;2.18) | 0.043 |
|  | rs2498018 | 1.49 | (1.01;2.18) | 0.043 |
|  | rs2504846 | 1.48 | (1.01;2.17) | 0.045 |
|  | rs252991 | 1.50 | (1.02;2.2) | 0.039 |
|  | rs2535692 | 1.51 | (1.03;2.21) | 0.037 |
|  | rs2570492 | 1.48 | (1.01;2.17) | 0.046 |
|  | rs2610990 | 1.52 | (1.03;2.22) | 0.034 |
|  | rs2624841 | 1.48 | (1.01;2.18) | 0.045 |
|  | rs2630767 | 1.49 | (1.02;2.19) | 0.041 |
|  | rs2631535 | 1.49 | (1.01;2.18) | 0.043 |
|  | rs2639655 | 1.49 | (1.01;2.18) | 0.042 |
|  | rs2668196 | 1.49 | (1.01;2.18) | 0.042 |
|  | rs273028 | 1.50 | (1.02;2.2) | 0.039 |
|  | rs2764684 | 1.50 | (1.02;2.21) | 0.038 |
|  | rs28381527 | 1.50 | (1.02;2.21) | 0.037 |
|  | rs2841425 | 1.50 | (1.02;2.21) | 0.038 |
|  | rs28438078 | 1.51 | (1.03;2.22) | 0.036 |
|  | rs28458909 | 1.52 | (1.03;2.23) | 0.033 |
|  | rs28588750 | 1.48 | (1.01;2.18) | 0.044 |
|  | rs28617748 | 1.51 | (1.03;2.22) | 0.036 |
|  | rs28807201 | 1.50 | (1.02;2.21) | 0.038 |
|  | rs2884364 | 1.51 | (1.03;2.21) | 0.037 |
|  | rs2901785 | 1.52 | (1.04;2.23) | 0.032 |
|  | rs2903624 | 1.51 | (1.03;2.22) | 0.034 |
|  | rs2916490 | 1.50 | (1.02;2.2) | 0.039 |
|  | rs2964252 | 1.50 | (1.02;2.2) | 0.038 |
|  | rs2974337 | 1.50 | (1.02;2.21) | 0.038 |
|  | rs3013170 | 1.49 | (1.02;2.19) | 0.040 |
|  | rs303752 | 1.49 | (1.01;2.18) | 0.042 |
|  | rs3095075 | 1.51 | (1.03;2.22) | 0.036 |
|  | rs3098650 | 1.51 | (1.03;2.21) | 0.036 |
|  | rs3108680 | 1.50 | (1.02;2.21) | 0.038 |
|  | rs329120 | 1.52 | (1.04;2.24) | 0.032 |
|  | rs336433 | 1.50 | (1.02;2.21) | 0.038 |
|  | rs34305371 | 1.45 | (0.99;2.14) | 0.056 |
|  | rs34316 | 1.50 | (1.02;2.21) | 0.038 |
|  | rs34488670 | 1.49 | (1.02;2.19) | 0.041 |
|  | rs34719425 | 1.50 | (1.02;2.2) | 0.038 |
|  | rs34743418 | 1.50 | (1.02;2.2) | 0.040 |
|  | rs34984805 | 1.50 | (1.02;2.2) | 0.040 |
|  | rs35226705 | 1.51 | (1.03;2.21) | 0.036 |
|  | rs35319653 | 1.48 | (1.01;2.18) | 0.044 |
|  | rs35375125 | 1.52 | (1.04;2.23) | 0.032 |
|  | rs35414043 | 1.49 | (1.01;2.18) | 0.043 |
|  | rs35414759 | 1.48 | (1.01;2.18) | 0.043 |
|  | rs35565533 | 1.49 | (1.02;2.18) | 0.042 |
|  | rs35583726 | 1.50 | (1.02;2.21) | 0.038 |
|  | rs356903 | 1.51 | (1.03;2.21) | 0.036 |
|  | rs35733856 | 1.49 | (1.01;2.18) | 0.043 |
|  | rs35951883 | 1.51 | (1.03;2.21) | 0.036 |
|  | rs36048136 | 1.51 | (1.03;2.21) | 0.036 |
|  | rs3731507 | 1.48 | (1.01;2.18) | 0.044 |
|  | rs3747631 | 1.48 | (1.01;2.17) | 0.047 |
|  | rs3783006 | 1.51 | (1.03;2.22) | 0.035 |
|  | rs378342 | 1.51 | (1.03;2.21) | 0.036 |
|  | rs3796432 | 1.51 | (1.03;2.22) | 0.035 |
|  | rs382196 | 1.50 | (1.02;2.2) | 0.039 |
|  | rs3863241 | 1.51 | (1.03;2.22) | 0.034 |
|  | rs3869097 | 1.51 | (1.03;2.21) | 0.037 |
|  | rs4076457 | 1.53 | (1.04;2.24) | 0.031 |
|  | rs4127499 | 1.50 | (1.02;2.2) | 0.040 |
|  | rs4141463 | 1.48 | (1.01;2.17) | 0.045 |
|  | rs41741 | 1.49 | (1.01;2.18) | 0.042 |
|  | rs421154 | 1.50 | (1.02;2.2) | 0.039 |
|  | rs42210 | 1.51 | (1.03;2.22) | 0.036 |
|  | rs422247 | 1.50 | (1.02;2.21) | 0.038 |
|  | rs424387 | 1.50 | (1.02;2.2) | 0.038 |
|  | rs4279337 | 1.51 | (1.03;2.22) | 0.036 |
|  | rs4358081 | 1.49 | (1.01;2.18) | 0.043 |
|  | rs4375600 | 1.51 | (1.03;2.21) | 0.037 |
|  | rs4390219 | 1.49 | (1.02;2.19) | 0.040 |
|  | rs4391653 | 1.51 | (1.03;2.22) | 0.036 |
|  | rs4409028 | 1.50 | (1.02;2.2) | 0.039 |
|  | rs4447403 | 1.50 | (1.02;2.21) | 0.037 |
|  | rs4467547 | 1.53 | (1.04;2.24) | 0.031 |
|  | rs4551987 | 1.51 | (1.03;2.22) | 0.036 |
|  | rs4557790 | 1.50 | (1.02;2.21) | 0.038 |
|  | rs4583487 | 1.53 | (1.04;2.25) | 0.031 |
|  | rs4588749 | 1.49 | (1.02;2.19) | 0.041 |
|  | rs4650228 | 1.50 | (1.02;2.21) | 0.038 |
|  | rs4663617 | 1.50 | (1.02;2.2) | 0.038 |
|  | rs4667878 | 1.50 | (1.02;2.2) | 0.039 |
|  | rs4679353 | 1.50 | (1.02;2.2) | 0.040 |
|  | rs4685448 | 1.49 | (1.01;2.18) | 0.042 |
|  | rs4726070 | 1.50 | (1.02;2.2) | 0.039 |
|  | rs474000 | 1.49 | (1.01;2.18) | 0.043 |
|  | rs4741600 | 1.51 | (1.03;2.22) | 0.035 |
|  | rs4751360 | 1.51 | (1.03;2.21) | 0.037 |
|  | rs4766975 | 1.50 | (1.02;2.2) | 0.040 |
|  | rs4772268 | 1.49 | (1.02;2.19) | 0.041 |
|  | rs4788080 | 1.49 | (1.02;2.19) | 0.041 |
|  | rs4798783 | 1.50 | (1.02;2.2) | 0.039 |
|  | rs4805761 | 1.50 | (1.02;2.21) | 0.037 |
|  | rs4812818 | 1.51 | (1.03;2.22) | 0.035 |
|  | rs4818226 | 1.48 | (1.01;2.18) | 0.044 |
|  | rs4846724 | 1.48 | (1.01;2.18) | 0.043 |
|  | rs4850810 | 1.54 | (1.05;2.26) | 0.026 |
|  | rs4864881 | 1.50 | (1.02;2.2) | 0.039 |
|  | rs4869579 | 1.49 | (1.02;2.19) | 0.041 |
|  | rs4869852 | 1.50 | (1.02;2.2) | 0.039 |
|  | rs4876775 | 1.49 | (1.02;2.19) | 0.041 |
|  | rs4879832 | 1.50 | (1.02;2.2) | 0.040 |
|  | rs4882141 | 1.52 | (1.04;2.23) | 0.032 |
|  | rs4883624 | 1.51 | (1.03;2.22) | 0.035 |
|  | rs489408 | 1.50 | (1.02;2.21) | 0.037 |
|  | rs490535 | 1.50 | (1.02;2.2) | 0.039 |
|  | rs4911257 | 1.49 | (1.02;2.19) | 0.041 |
|  | rs4989480 | 1.51 | (1.03;2.22) | 0.035 |
|  | rs5021426 | 1.53 | (1.05;2.25) | 0.028 |
|  | rs55640000 | 1.51 | (1.03;2.21) | 0.036 |
|  | rs55897719 | 1.51 | (1.03;2.21) | 0.036 |
|  | rs55975662 | 1.50 | (1.02;2.2) | 0.040 |
|  | rs56016333 | 1.52 | (1.03;2.23) | 0.034 |
|  | rs56059718 | 1.53 | (1.04;2.24) | 0.030 |
|  | rs56133711 | 1.51 | (1.03;2.21) | 0.037 |
|  | rs561655 | 1.52 | (1.03;2.23) | 0.033 |
|  | rs56188374 | 1.51 | (1.03;2.22) | 0.036 |
|  | rs56194430 | 1.49 | (1.02;2.19) | 0.041 |
|  | rs56306348 | 1.49 | (1.02;2.19) | 0.041 |
|  | rs56355837 | 1.51 | (1.03;2.22) | 0.036 |
|  | rs56378598 | 1.51 | (1.03;2.21) | 0.037 |
|  | rs56409354 | 1.52 | (1.03;2.23) | 0.034 |
|  | rs565960 | 1.48 | (1.01;2.18) | 0.044 |
|  | rs567775 | 1.48 | (1.01;2.17) | 0.043 |
|  | rs57349798 | 1.50 | (1.02;2.2) | 0.040 |
|  | rs5759002 | 1.50 | (1.02;2.21) | 0.037 |
|  | rs5765717 | 1.50 | (1.02;2.2) | 0.038 |
|  | rs586829 | 1.52 | (1.03;2.23) | 0.033 |
|  | rs58694847 | 1.50 | (1.02;2.2) | 0.039 |
|  | rs590013 | 1.49 | (1.02;2.19) | 0.042 |
|  | rs59903549 | 1.49 | (1.02;2.18) | 0.042 |
|  | rs60053512 | 1.51 | (1.03;2.21) | 0.036 |
|  | rs6020560 | 1.49 | (1.01;2.19) | 0.042 |
|  | rs6028084 | 1.48 | (1;2.17) | 0.047 |
|  | rs60307735 | 1.49 | (1.01;2.18) | 0.043 |
|  | rs6044142 | 1.51 | (1.03;2.21) | 0.036 |
|  | rs60814418 | 1.55 | (1.06;2.27) | 0.024 |
|  | rs6088618 | 1.50 | (1.02;2.2) | 0.038 |
|  | rs6091534 | 1.49 | (1.02;2.19) | 0.040 |
|  | rs6093705 | 1.50 | (1.02;2.2) | 0.040 |
|  | rs61160187 | 1.52 | (1.04;2.24) | 0.032 |
|  | rs61747226 | 1.49 | (1.02;2.19) | 0.041 |
|  | rs61964892 | 1.50 | (1.02;2.2) | 0.038 |
|  | rs62079997 | 1.51 | (1.03;2.21) | 0.036 |
|  | rs62086577 | 1.52 | (1.03;2.22) | 0.033 |
|  | rs62114178 | 1.50 | (1.02;2.21) | 0.038 |
|  | rs62156740 | 1.50 | (1.02;2.21) | 0.038 |
|  | rs62172117 | 1.51 | (1.03;2.22) | 0.035 |
|  | rs62199988 | 1.50 | (1.02;2.21) | 0.038 |
|  | rs62244884 | 1.53 | (1.04;2.24) | 0.031 |
|  | rs62247449 | 1.51 | (1.03;2.21) | 0.037 |
|  | rs62262312 | 1.49 | (1.01;2.18) | 0.043 |
|  | rs623200 | 1.50 | (1.02;2.2) | 0.038 |
|  | rs62367470 | 1.51 | (1.03;2.21) | 0.036 |
|  | rs62439683 | 1.50 | (1.02;2.2) | 0.040 |
|  | rs62543169 | 1.50 | (1.02;2.2) | 0.039 |
|  | rs6428152 | 1.50 | (1.02;2.2) | 0.038 |
|  | rs6440854 | 1.50 | (1.02;2.2) | 0.039 |
|  | rs6457796 | 1.51 | (1.03;2.22) | 0.035 |
|  | rs6466819 | 1.53 | (1.05;2.25) | 0.029 |
|  | rs6499084 | 1.50 | (1.02;2.2) | 0.038 |
|  | rs6502670 | 1.50 | (1.02;2.21) | 0.038 |
|  | rs6534338 | 1.47 | (1;2.15) | 0.049 |
|  | rs6534704 | 1.51 | (1.03;2.21) | 0.037 |
|  | rs6539284 | 1.50 | (1.02;2.2) | 0.039 |
|  | rs6545559 | 1.50 | (1.02;2.2) | 0.039 |
|  | rs6547396 | 1.49 | (1.02;2.19) | 0.041 |
|  | rs6563363 | 1.51 | (1.03;2.22) | 0.034 |
|  | rs6580068 | 1.51 | (1.02;2.21) | 0.037 |
|  | rs660010 | 1.50 | (1.02;2.2) | 0.040 |
|  | rs66671632 | 1.51 | (1.03;2.21) | 0.036 |
|  | rs669952 | 1.51 | (1.03;2.21) | 0.036 |
|  | rs67033024 | 1.50 | (1.02;2.21) | 0.037 |
|  | rs67040074 | 1.50 | (1.02;2.21) | 0.038 |
|  | rs6704241 | 1.50 | (1.02;2.2) | 0.038 |
|  | rs6704768 | 1.53 | (1.04;2.24) | 0.031 |
|  | rs6713695 | 1.51 | (1.03;2.22) | 0.035 |
|  | rs6715321 | 1.52 | (1.04;2.24) | 0.031 |
|  | rs6721505 | 1.51 | (1.03;2.22) | 0.035 |
|  | rs6731373 | 1.49 | (1.02;2.19) | 0.041 |
|  | rs6747129 | 1.48 | (1.01;2.17) | 0.046 |
|  | rs6779981 | 1.50 | (1.02;2.21) | 0.037 |
|  | rs6782698 | 1.50 | (1.02;2.21) | 0.038 |
|  | rs67829508 | 1.50 | (1.02;2.2) | 0.040 |
|  | rs6801153 | 1.50 | (1.02;2.21) | 0.037 |
|  | rs6839051 | 1.52 | (1.03;2.22) | 0.033 |
|  | rs6898748 | 1.50 | (1.02;2.2) | 0.039 |
|  | rs6907381 | 1.49 | (1.02;2.19) | 0.042 |
|  | rs6911986 | 1.49 | (1.02;2.19) | 0.041 |
|  | rs6929638 | 1.49 | (1.02;2.19) | 0.041 |
|  | rs6930903 | 1.53 | (1.04;2.25) | 0.030 |
|  | rs6956241 | 1.48 | (1.01;2.17) | 0.045 |
|  | rs6960056 | 1.50 | (1.02;2.2) | 0.039 |
|  | rs7009856 | 1.50 | (1.02;2.2) | 0.039 |
|  | rs7026972 | 1.49 | (1.02;2.19) | 0.041 |
|  | rs7027019 | 1.50 | (1.02;2.2) | 0.039 |
|  | rs7027899 | 1.50 | (1.02;2.21) | 0.037 |
|  | rs7032484 | 1.52 | (1.03;2.23) | 0.033 |
|  | rs7039819 | 1.51 | (1.03;2.22) | 0.035 |
|  | rs705240 | 1.51 | (1.03;2.22) | 0.034 |
|  | rs708912 | 1.50 | (1.02;2.2) | 0.040 |
|  | rs7097348 | 1.50 | (1.02;2.2) | 0.039 |
|  | rs7109373 | 1.51 | (1.03;2.22) | 0.036 |
|  | rs7131691 | 1.52 | (1.04;2.23) | 0.031 |
|  | rs71366589 | 1.50 | (1.02;2.19) | 0.040 |
|  | rs7150195 | 1.50 | (1.02;2.21) | 0.037 |
|  | rs7151326 | 1.51 | (1.03;2.22) | 0.035 |
|  | rs7172133 | 1.51 | (1.03;2.22) | 0.035 |
|  | rs717997 | 1.50 | (1.02;2.21) | 0.038 |
|  | rs7184863 | 1.50 | (1.02;2.2) | 0.039 |
|  | rs7236339 | 1.49 | (1.02;2.19) | 0.041 |
|  | rs7240432 | 1.51 | (1.03;2.22) | 0.035 |
|  | rs7254263 | 1.49 | (1.01;2.18) | 0.043 |
|  | rs72672601 | 1.49 | (1.01;2.19) | 0.042 |
|  | rs72674843 | 1.50 | (1.02;2.2) | 0.039 |
|  | rs72695265 | 1.50 | (1.02;2.2) | 0.040 |
|  | rs72717150 | 1.50 | (1.02;2.2) | 0.040 |
|  | rs72725852 | 1.50 | (1.02;2.2) | 0.040 |
|  | rs72748123 | 1.51 | (1.03;2.22) | 0.035 |
|  | rs72778512 | 1.50 | (1.02;2.21) | 0.037 |
|  | rs72801843 | 1.50 | (1.02;2.2) | 0.039 |
|  | rs72819955 | 1.50 | (1.02;2.2) | 0.039 |
|  | rs72829857 | 1.50 | (1.02;2.21) | 0.038 |
|  | rs72838750 | 1.50 | (1.02;2.2) | 0.040 |
|  | rs72890842 | 1.51 | (1.03;2.22) | 0.036 |
|  | rs72906124 | 1.50 | (1.02;2.21) | 0.037 |
|  | rs72915557 | 1.50 | (1.02;2.21) | 0.037 |
|  | rs7296742 | 1.50 | (1.02;2.21) | 0.037 |
|  | rs72993796 | 1.50 | (1.02;2.21) | 0.038 |
|  | rs7301232 | 1.51 | (1.03;2.22) | 0.035 |
|  | rs7302808 | 1.49 | (1.02;2.19) | 0.042 |
|  | rs7304399 | 1.53 | (1.04;2.25) | 0.029 |
|  | rs73055556 | 1.51 | (1.03;2.22) | 0.036 |
|  | rs7306755 | 1.49 | (1.01;2.18) | 0.044 |
|  | rs73117392 | 1.50 | (1.02;2.21) | 0.038 |
|  | rs7312511 | 1.51 | (1.03;2.22) | 0.035 |
|  | rs73141547 | 1.49 | (1.02;2.19) | 0.041 |
|  | rs73210523 | 1.52 | (1.04;2.23) | 0.032 |
|  | rs73219806 | 1.50 | (1.02;2.2) | 0.040 |
|  | rs7328289 | 1.50 | (1.02;2.2) | 0.040 |
|  | rs7338804 | 1.50 | (1.02;2.21) | 0.038 |
|  | rs7356921 | 1.48 | (1.01;2.18) | 0.044 |
|  | rs738988 | 1.51 | (1.03;2.21) | 0.036 |
|  | rs7430651 | 1.49 | (1.01;2.19) | 0.042 |
|  | rs7449561 | 1.50 | (1.02;2.21) | 0.037 |
|  | rs74677779 | 1.50 | (1.02;2.21) | 0.037 |
|  | rs7469569 | 1.49 | (1.01;2.18) | 0.043 |
|  | rs74740938 | 1.50 | (1.02;2.2) | 0.039 |
|  | rs748832 | 1.51 | (1.03;2.22) | 0.035 |
|  | rs7515450 | 1.51 | (1.03;2.22) | 0.035 |
|  | rs7520927 | 1.50 | (1.02;2.21) | 0.038 |
|  | rs75624576 | 1.48 | (1.01;2.18) | 0.044 |
|  | rs7578247 | 1.51 | (1.03;2.22) | 0.035 |
|  | rs75795256 | 1.51 | (1.03;2.22) | 0.034 |
|  | rs7603132 | 1.51 | (1.02;2.21) | 0.037 |
|  | rs76076331 | 1.46 | (0.99;2.14) | 0.055 |
|  | rs7626224 | 1.50 | (1.02;2.2) | 0.039 |
|  | rs7632819 | 1.46 | (1;2.14) | 0.051 |
|  | rs7650602 | 1.51 | (1.03;2.22) | 0.035 |
|  | rs7668960 | 1.53 | (1.04;2.25) | 0.029 |
|  | rs76878669 | 1.51 | (1.03;2.21) | 0.037 |
|  | rs77128898 | 1.52 | (1.04;2.24) | 0.031 |
|  | rs77187837 | 1.50 | (1.02;2.21) | 0.037 |
|  | rs77584294 | 1.50 | (1.02;2.2) | 0.040 |
|  | rs7762296 | 1.50 | (1.02;2.2) | 0.039 |
|  | rs77882218 | 1.50 | (1.02;2.2) | 0.039 |
|  | rs7788620 | 1.53 | (1.04;2.24) | 0.031 |
|  | rs77899607 | 1.49 | (1.02;2.19) | 0.041 |
|  | rs77926719 | 1.50 | (1.02;2.2) | 0.039 |
|  | rs78116078 | 1.51 | (1.03;2.21) | 0.037 |
|  | rs78280128 | 1.52 | (1.04;2.24) | 0.032 |
|  | rs78410329 | 1.50 | (1.02;2.21) | 0.038 |
|  | rs78462816 | 1.51 | (1.03;2.21) | 0.037 |
|  | rs786244 | 1.48 | (1.01;2.17) | 0.045 |
|  | rs792213 | 1.50 | (1.02;2.2) | 0.039 |
|  | rs7924036 | 1.49 | (1.02;2.19) | 0.042 |
|  | rs7924465 | 1.50 | (1.02;2.21) | 0.038 |
|  | rs7933521 | 1.50 | (1.02;2.19) | 0.040 |
|  | rs7936656 | 1.51 | (1.03;2.22) | 0.035 |
|  | rs795540 | 1.51 | (1.03;2.21) | 0.036 |
|  | rs7977614 | 1.50 | (1.02;2.2) | 0.039 |
|  | rs7979979 | 1.49 | (1.02;2.19) | 0.042 |
|  | rs7983020 | 1.51 | (1.03;2.22) | 0.035 |
|  | rs79925479 | 1.50 | (1.02;2.21) | 0.037 |
|  | rs80049472 | 1.51 | (1.03;2.21) | 0.037 |
|  | rs80171383 | 1.50 | (1.02;2.2) | 0.039 |
|  | rs8020023 | 1.50 | (1.02;2.2) | 0.038 |
|  | rs80257979 | 1.50 | (1.02;2.2) | 0.039 |
|  | rs80352808 | 1.51 | (1.03;2.21) | 0.036 |
|  | rs8044562 | 1.52 | (1.03;2.23) | 0.034 |
|  | rs8058137 | 1.49 | (1.01;2.19) | 0.042 |
|  | rs8072494 | 1.51 | (1.03;2.22) | 0.034 |
|  | rs8110747 | 1.50 | (1.02;2.2) | 0.039 |
|  | rs837080 | 1.49 | (1.02;2.19) | 0.042 |
|  | rs853199 | 1.50 | (1.02;2.2) | 0.039 |
|  | rs863006 | 1.50 | (1.02;2.2) | 0.039 |
|  | rs872602 | 1.51 | (1.03;2.22) | 0.036 |
|  | rs899223 | 1.50 | (1.02;2.2) | 0.040 |
|  | rs903981 | 1.50 | (1.02;2.2) | 0.039 |
|  | rs912609 | 1.50 | (1.02;2.2) | 0.039 |
|  | rs9317202 | 1.51 | (1.03;2.21) | 0.037 |
|  | rs9356034 | 1.50 | (1.02;2.21) | 0.038 |
|  | rs9359939 | 1.50 | (1.02;2.21) | 0.038 |
|  | rs9372734 | 1.45 | (0.99;2.13) | 0.058 |
|  | rs9374194 | 1.51 | (1.03;2.22) | 0.034 |
|  | rs9388490 | 1.49 | (1.01;2.18) | 0.043 |
|  | rs9411336 | 1.49 | (1.01;2.18) | 0.043 |
|  | rs9461242 | 1.49 | (1.01;2.19) | 0.043 |
|  | rs9496630 | 1.51 | (1.03;2.22) | 0.035 |
|  | rs952062 | 1.50 | (1.02;2.2) | 0.038 |
|  | rs9537509 | 1.50 | (1.02;2.2) | 0.039 |
|  | rs9540731 | 1.50 | (1.02;2.21) | 0.037 |
|  | rs9540920 | 1.50 | (1.03;2.21) | 0.037 |
|  | rs9551887 | 1.49 | (1.02;2.19) | 0.041 |
|  | rs9555651 | 1.50 | (1.02;2.2) | 0.039 |
|  | rs9635366 | 1.53 | (1.04;2.25) | 0.029 |
|  | rs9652254 | 1.49 | (1.01;2.18) | 0.042 |
|  | rs975303 | 1.53 | (1.04;2.24) | 0.030 |
|  | rs9787076 | 1.51 | (1.02;2.21) | 0.037 |
|  | rs9859719 | 1.51 | (1.03;2.21) | 0.036 |
|  | rs9871964 | 1.52 | (1.04;2.23) | 0.032 |
|  | rs9899056 | 1.51 | (1.03;2.21) | 0.036 |
|  | rs989996 | 1.50 | (1.02;2.21) | 0.037 |
|  | rs9914251 | 1.50 | (1.02;2.2) | 0.039 |
|  | rs9922788 | 1.51 | (1.03;2.22) | 0.035 |
|  | rs9930253 | 1.50 | (1.02;2.2) | 0.039 |
|  | rs9937449 | 1.51 | (1.03;2.22) | 0.034 |
|  | rs9985296 | 1.51 | (1.03;2.21) | 0.036 |
|  | rs9993133 | 1.50 | (1.02;2.2) | 0.040 |
|  | All | 1.50 | (1.02;2.2) | 0.038 |

**References**

Ghorbani Mojarrad, N., Plotnikov, D., Williams, C., Guggenheim, J. A., Eye, f. t. U. B., & Consortium, V. (2020). Association Between Polygenic Risk Score and Risk of Myopia. *JAMA Ophthalmology, 138*(1), 7-13. doi:10.1001/jamaophthalmol.2019.4421

Kurki, M. I., Karjalainen, J., Palta, P., Sipilä, T. P., Kristiansson, K., Donner, K. M., . . . FinnGen. (2023). FinnGen provides genetic insights from a well-phenotyped isolated population. *Nature, 613*(7944), 508-518. doi:10.1038/s41586-022-05473-8

Liu, M., Jiang, Y., Wedow, R., Li, Y., Brazel, D. M., Chen, F., . . . Psychiatry, H. A.-I. (2019). Association studies of up to 1.2 million individuals yield new insights into the genetic etiology of tobacco and alcohol use. *Nature Genetics, 51*(2), 237-244. doi:10.1038/s41588-018-0307-5

Okbay, A., Wu, Y., Wang, N., Jayashankar, H., Bennett, M., Nehzati, S. M., . . . LifeLines Cohort, S. (2022). Polygenic prediction of educational attainment within and between families from genome-wide association analyses in 3 million individuals. *Nature Genetics, 54*(4), 437-449. doi:10.1038/s41588-022-01016-z

Pulit, S. L., Stoneman, C., Morris, A. P., Wood, A. R., Glastonbury, C. A., Tyrrell, J., . . . Lindgren, C. M. (2018). Meta-analysis of genome-wide association studies for body fat distribution in 694 649 individuals of European ancestry. *Human Molecular Genetics, 28*(1), 166-174. doi:10.1093/hmg/ddy327
